# A viroid-derived system to produce large amounts of recombinant RNA in *Escherichia coli*

**DOI:** 10.1038/s41598-018-20314-3

**Published:** 2018-01-30

**Authors:** José-Antonio Daròs, Verónica Aragonés, Teresa Cordero

**Affiliations:** Instituto de Biología Molecular y Celular de Plantas (Consejo Superior de Investigaciones Científicas-Universitat Politècnica de València), 46022 Valencia, Spain

## Abstract

Viruses have been engineered into useful biotechnological tools for gene therapy or to induce the synthesis of products of interest, such as therapeutic proteins and vaccines, in animal and fungal cells, bacteria or plants. Viroids are a particular class of infectious agents of higher plants that exclusively consist of a small non-protein-coding circular RNA molecule. In the same way as viruses have been transformed into useful biotechnological devices, can viroids be converted into beneficial tools? We show herein that, by expressing *Eggplant latent viroid* (ELVd) derived RNAs in *Escherichia coli* together with the eggplant tRNA ligase, this being the enzyme involved in viroid circularization in the infected plant, RNAs of interest like aptamers, extended hairpins, or other structured RNAs are produced in amounts of tens of milligrams per liter of culture. Although ELVd fails to replicate in *E. coli*, ELVd precursors self-cleave through the embedded hammerhead ribozymes and the resulting monomers are, in part, circularized by the co-expressed enzyme. The mature viroid forms and the protein likely form a ribonucleoprotein complex that transitorily accumulates in *E. coli* cells at extraordinarily amounts.

## Introduction

The relatively small genomes of viroids, which range between 246 and 401 nucleotides (nt) in presently known species^1^, replicate in infected plant cells by an RNA-to-RNA rolling circle mechanism that consists of three steps: (i) synthesis of multimeric RNA transcripts of both polarities, (ii) cleavage of some of these transcripts to monomeric length, and (iii) ligation of these monomers to produce circular molecules^2–4^. Viroid replication is mediated by host enzymes, such as RNA polymerases^5,6^, RNases^7^ and ligases^8,9^, but also by the activity of the hammerhead ribozymes embedded in the RNAs of some viroid species^10,11^. More than 30 viroid species have been characterized to date and classified into two families, *Pospiviroidae* and *Avsunviroidae*^1^. Most viroids, like *Potato spindle tuber viroid* (PSTVd), contain a central conserved region (CCR), approximately in the middle of the folded molecule, and replicate in the nucleus of infected cells through the asymmetric version of the rolling circle mechanism. They belong to the family *Pospiviroidae*. In contrast, some viroids, like *Avocado sunblotch viroid* (ASBVd; type member of the family) or *Eggplant latent viroid* (ELVd)^12^, lack CCR, contain hammerhead ribozymes in the strands of both polarities, and replicate through the symmetric variant of the rolling circle mechanism in the chloroplasts of infected cells. They are classified into the family *Avsunviroidae*.

We recently demonstrated that, in the last step of PSTVd replication, the host DNA ligase 1 is redirected to accept RNA templates to circularize monomeric linear PSTVd RNAs^8^. For the viroids of the family *Avsunviroidae*, we also showed that the ELVd monomeric linear RNAs that result from ribozyme processing and contain 5′-hydroxyl and 2′,3′-cyclic phosphodiester termini are efficiently circularized *in vitro* by the chloroplastic isoform of eggplant tRNA ligase, and also that this reaction most probably occurs *in vivo*^9^. To further investigate the interaction between eggplant tRNA ligase and ELVd RNA, we co-expressed both molecules in *Escherichia coli*. Interestingly, when we analyzed the RNAs that accumulated in transformed bacteria, we observed that some ELVd RNAs accumulated at levels higher than the *E. coli* ribosomal RNAs did. This occurred despite the analysis of the ELVd-derived RNAs that accumulated in *E. coli* ruled out viroid replication in bacterial cells, because no replication intermediates were detected. This observation suggested that, in the presence of eggplant tRNA ligase, ELVd longer-than-unit transcripts are efficiently transcribed, cleaved and circularized, and that extraordinary levels of the resulting monomers transitorily accumulate in *E. coli* cells. Based on this observation, we aimed to develop a viroid-derived system to produce large amounts of recombinant RNAs in *E. coli*. This system is herein described and used to produce tens of milligrams of RNAs of interest per liter of *E. coli* culture, such as the RNA aptamer Spinach, which mimics fluorescent proteins^13^, hairpin RNAs like those that efficiently induce RNA silencing^14^, or the direct repeat-containing precursor of a CRISPR RNA (crRNA) like those that guide Cpf1 nuclease for genome editing^15^.

## Results

### The co-expression of ELVd RNA and eggplant tRNA ligase in *E. coli* induces accumulation of notable amounts of circular viroid monomers

**Figure 1:**
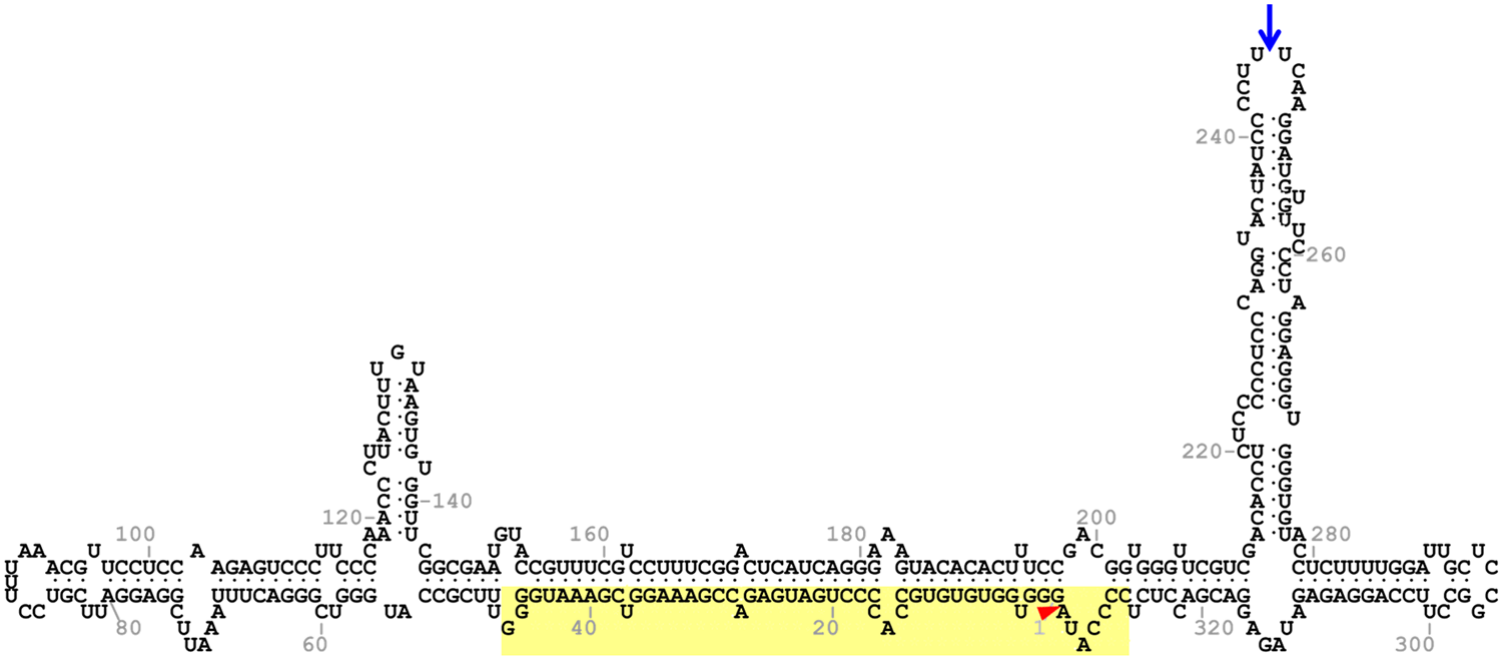
ELVd (sequence variant AJ536613) folded in the predicted secondary structure of minimum free energy. The domain and self-cleavage site of the hammerhead ribozyme are indicated on yellow background and by a red arrowhead, respectively. The internucleotide position U245-U246 where the RNAs of interest were grafted is indicated by a blue arrow.

**Figure 2:**
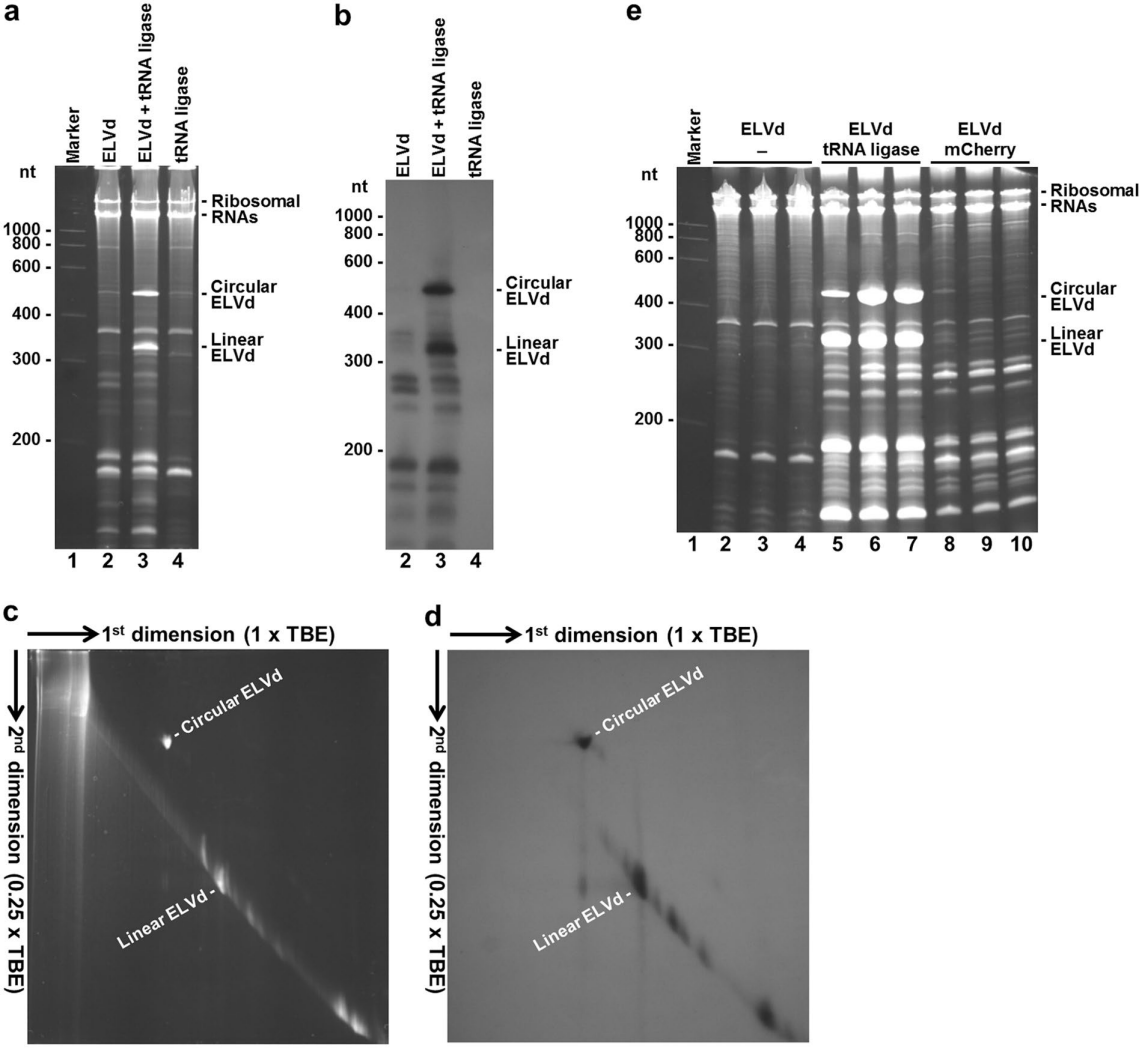
Analysis of RNAs accumulated in *E. coli* cells that co-express ELVd RNA and eggplant tRNA ligase. RNAs were separated by PAGE, stained first with ethidium bromide (**a**,**c** and **e**), and transferred next to membranes, hybridized with a radioactive probe complementary to ELVd RNA and autoradiographed (**b** and **d**). (**a** and **b**) RNAs separated by single denaturing PAGE. Lane 1, RNA marker with the sizes of the standards indicated on the left in nt; lanes 2 to 4, RNAs from the *E. coli* cells transformed with pLELVd (lane 2), pLELVd and p15tRnlSm (lane 3) and p15tRnlSm (lane 4). (**c** and **d**) RNAs from the *E. coli* co-transformed with pLELVd and p15tRnlSm separated by two-dimension denaturing PAGE in two different buffer conditions, as indicated. (**e**) RNAs separated by single denaturing PAGE. Lane 1, RNA marker with the sizes of the standards indicated on the left in nt; lanes 2 to 10, RNAs from independent *E. coli* clones transformed with pLELVd (lanes 2 to 4), pLELVd and p15tRnlSm (lanes 5 to 7), and pLELVd and p15mCherry (lanes 8 to 10). In the different panels, the positions of the circular and linear ELVd RNAs, and of *E. coli* 23S and 16S rRNAs, are indicated. (**a**–**d**) *E. coli* cultures were grown at 25 °C and induced with 0.4 mM IPTG at OD_600_ 0.6. Bacteria were harvested at 10 h post-induction. (**e**) *E. coli* cultures were grown at 37 °C and induced with 0.1 mM IPTG at OD_600_ 0.1. Bacteria were harvested at 8 h post-induction. Each lane contains an aliquot of RNA that corresponds to 0.8 ml of culture in all cases.

To investigate the domains and residues in both the ELVd RNA (Fig. 1) and tRNA ligase involved in viroid circularization, we set up an experimental system that consisted of recombinant *E. coli* clones transformed with plasmids to co-express both molecules (pLELVd and p15tRnlSm, Supplementary Fig. S1, see Supplementary Information). Longer-than-unit ELVd RNA (from C327 to G46, GenBank accession number AJ536613; note that ELVd is circular and A333 is followed by G1), which included the repetition of the plus-strand hammerhead ribozyme domain (Fig. 1), was constitutively expressed under the control of the *E. coli* murein lipoprotein promoter and the 5S rRNA (*rrnC*) terminator. A recombinant version of eggplant tRNA ligase, which included the amino-terminal transit peptide that mediates translocation to chloroplasts^16^, but also a carboxy-terminal hexahistidine tag^9^, was expressed in a compatible plasmid of p15A replication origin under the control of the T7 bacteriophage promoter and terminator. The tRNA ligase expression was induced in cultures of *E. coli* strain BL21(DE3) by adding isopropyl β-D-1-thiogalactopyranoside (IPTG), which activated the expression of a copy of bacteriophage T7 RNA polymerase set under the control of the lacUV5 promoter. *E. coli* cultures were grown at 25 °C to an optic density at 600 nm (OD_600_) of 0.6 and the tRNA ligase expression was induced by adding IPTG to 0.4 mM. Cells were harvested at 10 h post-induction and total RNA was recovered by phenol:chloroform extraction. Aliquots of the RNA preparations were separated by denaturing (8 M urea) polyacrylamide gel electrophoresis (PAGE). Gels were first stained with ethidium bromide, and then RNA was blotted to membranes and analyzed by northern blot hybridization using an ELVd ^32^P-labeled RNA probe of complementary polarity. This analysis showed that the bacteria co-transformed with plasmids to express ELVd RNA and eggplant tRNA ligase accumulated substantial amounts of the viroid monomeric circular and linear forms, which were clearly detected in both the stained gel (Fig. 2a, lane 3) and the hybridized membrane (Fig. 2b, lane 3). These particular viroid species hardly accumulate in the bacteria transformed with a plasmid to express ELVd RNA alone (Fig. 2b, lane 2). In contrast, species that migrated faster than the monomeric linear ELVd RNA and that most probably correspond to ELVd degradation products were detected in both samples (Fig. 2b, lanes 2 and 3). No ELVd was detected in control bacteria transformed with a plasmid to express the tRNA ligase alone (Fig. 2b, lane 4). The circular nature of the slow-migrating species was confirmed by separating RNA by two-dimension PAGE (Fig. 2c and d). The very minor amounts of monomeric circular ELVd RNA detected in the absence of tRNA ligase (Fig. 2b, lane 2) most probably result from the 2′,5′ self-ligation reaction previously described in this viroid family^17,18^.

**Figure 3:**
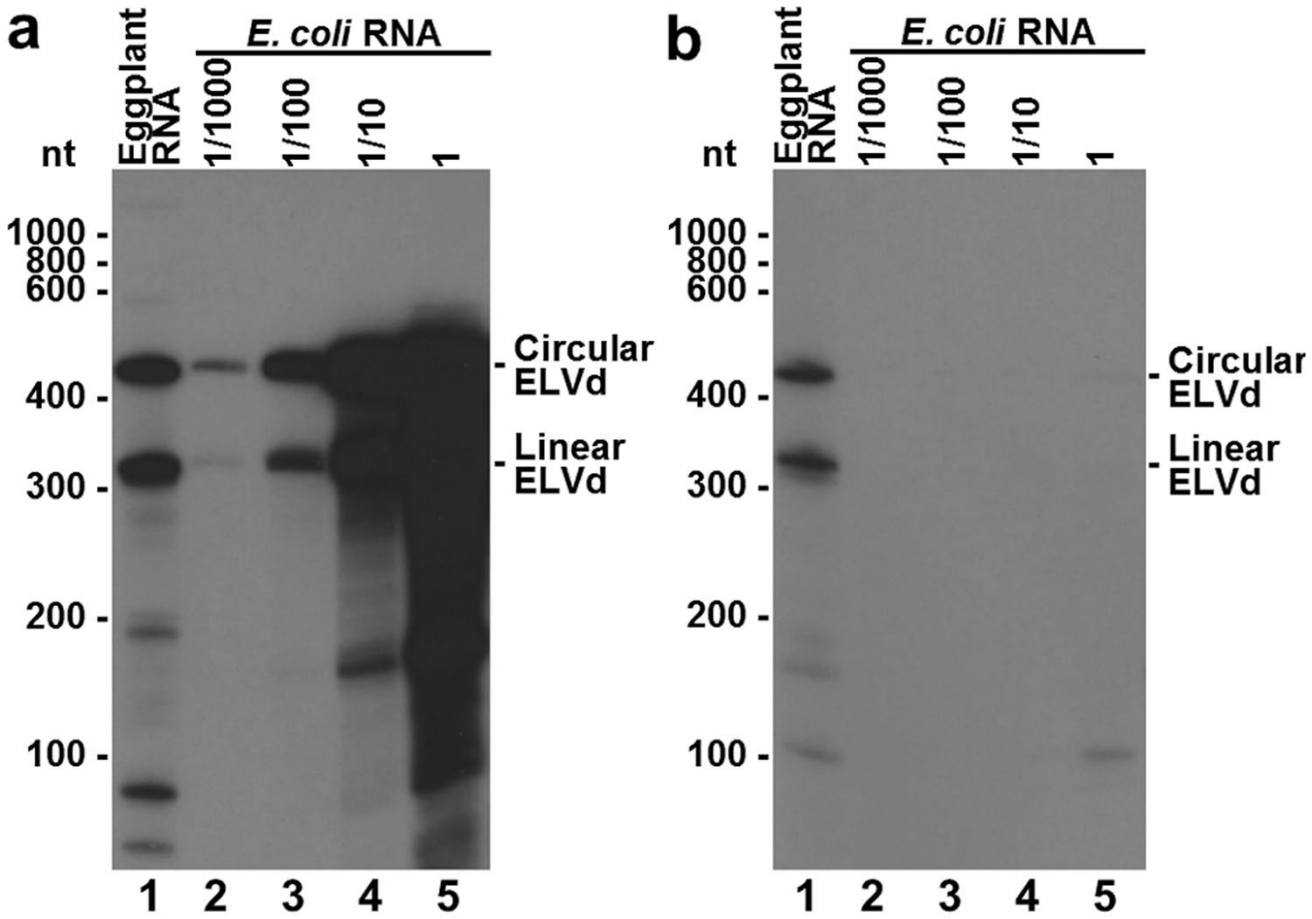
Polarity analysis of the ELVd RNAs that accumulated in *E. coli* cells that co-express ELVd RNA and eggplant tRNA ligase. RNAs were separated by PAGE, stained with ethidium bromide, transferred to membranes and hybridized with RNA radioactive probes to detect ELVd RNAs of plus (**a**) and minus (**b**) polarities. Lane 1, total RNAs purified from an ELVd-infected eggplant; lanes 2 to 5, ELVd RNAs purified from *E. coli* cells transformed with pLELVd and p15tRnlSm. To better appreciate the differential hybridization with both ELVd probes, ELVd RNAs from *E. coli* (lane 5) were loaded serially diluted 1/10 in lane 4, 1/100 in lane 3 and 1/1000 in lane 2. The positions and sizes (in nt) of RNA markers are indicated on the left of both panels. The positions of monomeric circular and linear ELVd RNAs are indicated on the right of both panels.

These results suggested that accumulation of monomeric circular and linear ELVd RNAs in *E. coli* depended on the activity of the co-expressed eggplant tRNA ligase. To gain insight into this hypothesis, we constructed a new plasmid (p15mCherry, Supplementary Fig. S1) to express fluorescent protein mCherry^19^, herein taken as an easy-to-track heterologous control. Once again, only the cells that co-expressed ELVd RNA and tRNA ligase accumulated large amounts of monomeric circular and linear ELVd RNA (Fig. 2e, lanes 5 to 7). It is remarkable to note that, according to the intensity of the bands in the polyacrylamide gels, extraordinary levels of these viroid species accumulated, which were higher than, or at least similar to, those of *E. coli* 23S and 16S rRNAs (Fig. 2a and e). However, oligomeric ELVd RNAs (Fig. 2b and d) and viroid strands of opposite polarity (Fig. 3), typical intermediates of the rolling-circle replication mechanism, were never detected in the hybridization blots, which indicated that ELVd does not replicate in recombinant *E. coli* cells.

### Remarkable amounts of the RNAs of interest inserted into the ELVd molecule also accumulate in *E. coli* when co-expressed with tRNA ligase

The outstanding accumulation of the monomeric forms of ELVd in the *E. coli* cells, in which eggplant tRNA ligase was co-expressed, led us to consider developing a viroid-derived system to overproduce recombinant RNA. We hypothesized that since ELVd accumulation did not depend on viroid replication, RNAs of interest grafted onto the ELVd molecule would also be efficiently produced in *E. coli*. According to this aim, we first analyzed the culture condition that maximized ELVd production in *E. coli*. We compared ELVd accumulation in cultures grown at 25 °C and 37 °C. Although ELVd accumulation was transitory and the viroid almost disappeared with prolonged culture times, the cells grown at 37 °C accumulated more ELVd monomers than those grown at 25 °C (Supplementary Fig. S2). Within the range of 0.1 to 1.6 mM IPTG, the best ELVd production was observed in the cultures in which the tRNA ligase expression was induced at the lowest IPTG concentration (Supplementary Fig. S3). However, some IPTG was needed since the control with no added IPTG did not accumulate significant amounts of ELVd. This result reinforces the role of tRNA ligase in the ELVd accumulation process. An analysis of two time-course ELVd expressions, in which IPTG to 0.1 mM was added at cell OD_600_ of 0.6 or 0.1, showed better production in the second case (Supplementary Fig. S4). Remarkably, a comparison of the classic Luria-Bertani (LB) with richer Terrific Broth (TB) media to grow bacteria revealed that ELVd accumulated to extremely high levels in the *E. coli* cells grown in TB medium (Supplementary Fig. S5, see lane 15). A time-course experiment under the best experimental conditions (growth at 37 °C in TB medium with the tRNA ligase expression induced at OD_600_ 0.1 with 0.1 mM IPTG) showed that the amount of monomeric circular and linear ELVd RNAs per culture volume increased with time and reached a maximum at around 12 h post-induction, and then decreased to essentially disappear at 24 h (Supplementary Fig. S6). Comparing with an RNA standard, we estimated a total combined accumulation of 150 mg of monomeric circular and linear ELVd RNAs per liter of bacterial culture with a circular:linear ratio of 3:1 (Supplementary Fig. S7).

Next, we investigated the possibility of reducing the size of the ELVd moiety in the final recombinant product. To this end, we constructed a series of pLELVd-derived plasmids in which different viroid cDNA fragments were deleted (Supplementary Fig. S8). The procedure for the deletions consisted in sequentially removing different elements of the viroid secondary structure without affecting the hammerhead ribozyme domain, which mediates the self-cleavage of the primary transcript (Supplementary Fig. S9). We analyzed how single (Supplementary Fig. S10) and double (Supplementary Fig. S11) deletions affected the accumulation of the resulting ELVd forms in *E. coli*. We found that ELVd deleted forms L1R3, L3R1 and L2R2, which respectively consisted of 175, 215 and 246 nt, still accumulated efficiently in *E. coli* when tRNA ligase was co-expressed (Supplementary Fig. S11). Moreover, we reasoned that the need to induce the tRNA ligase expression by adding IPTG at a particular cell density can be a serious inconvenience for eventually scaling up the system to industrial fermenters, in which cells could be grown continuously. So in order to further optimize the system, we constructed a new plasmid (p15LtRnlSm) in which the T7 bacteriophage RNA polymerase promoter present in p15tRnlSm was replaced with the constitutive *E. coli* murein lipoprotein promoter (Supplementary Fig. S12). *E. coli* BL21(DE3) was co-transformed with pLELVd, and with either the original p15tRnlSm or new p15LtRnlSm. Recombinant clones were grown to OD_600_ 0.1 and were induced, or not, with IPTG depending on the harbored tRNA ligase plasmid. A time-course analysis of the RNA produced in both cultures showed no substantial difference in circular and linear ELVd RNA production (Supplementary Fig. S13).

**Figure 4:**
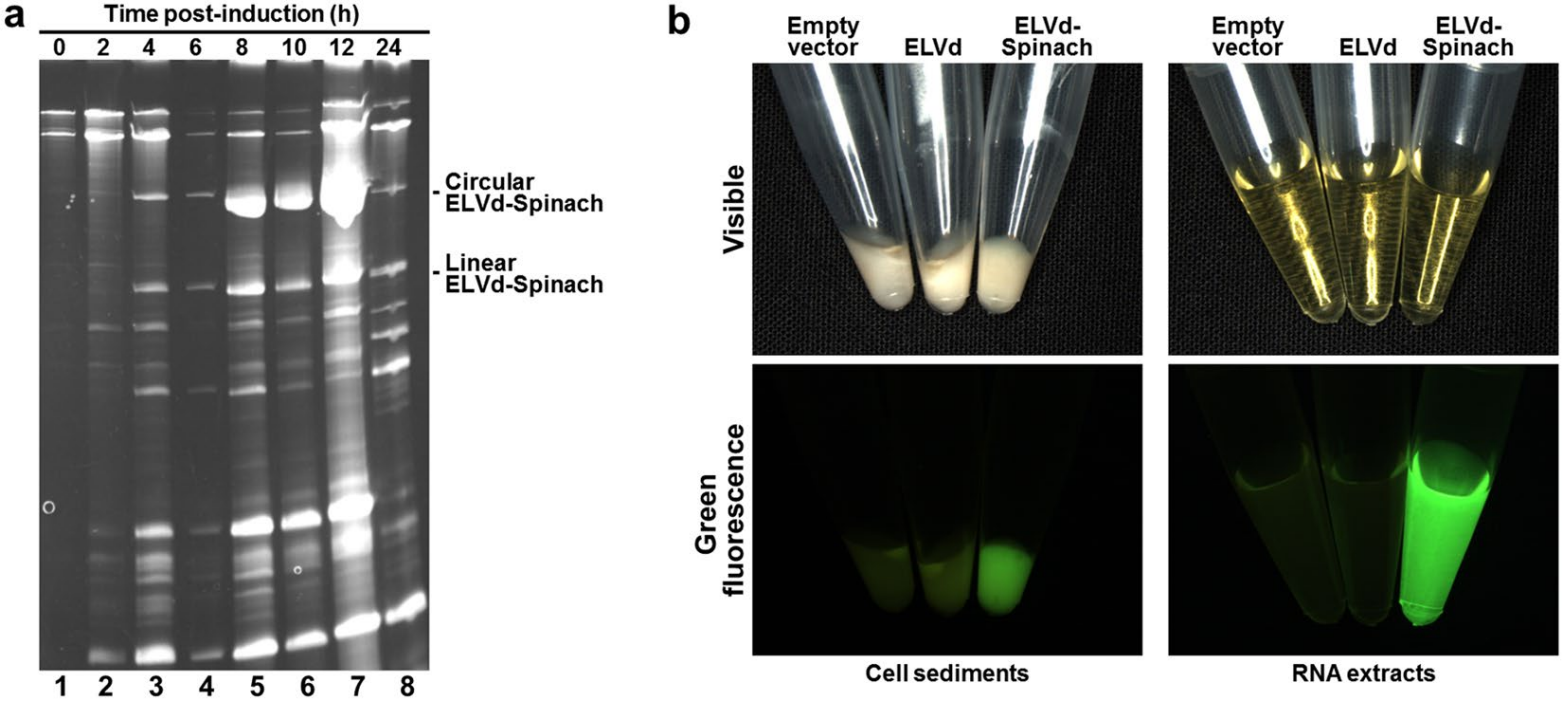
Recombinant ELVd-Spinach RNA production in *E. coli* using the viroid-derived system. (**a**) Time-course analysis of the RNA that accumulated in *E. coli* cells that co-expressed ELVd-Spinach RNA and eggplant tRNA ligase. Aliquots were taken from the culture at different time points and RNA was extracted. RNAs were separated by denaturing PAGE and the gel was stained with ethidium bromide. Lanes 1 to 8, RNAs from the aliquots taken at 0, 2, 4, 6, 8, 10, 12 and 24 h post-induction of tRNA ligase expression. The positions of the circular and linear ELVd-Spinach RNAs are indicated on the right. *E. coli* cultures were grown at 37 °C in TB medium and induced with 0.1 mM IPTG at OD_600_ 0.1 mM. Each lane contains the RNA that corresponds to 0.4 ml of culture. (**b**) Pictures of the sedimented *E. coli* cells that co-expressed tRNA ligase and an empty vector, ELVd or ELVd-Spinach, as indicated (left), and the corresponding RNA extracts (right). The lower pictures were taken under UV illumination using a GFP filter. DFHBI was added to either the *E. coli* culture before cell recovery (left) or the RNA extract (right).

Then, we assayed the production of RNAs of interest inserted into the ELVd molecule. We chose the terminal loop of the upper-right hairpin present in the hypothetical ELVd conformation of minimum free energy as the insertion site (positions U245-U246 of ELVd-AJ536613, Fig. 1). A previous mutational analysis of this viroid has demonstrated that an insertion of eight nucleotides into this particular position, unlike other assayed positions, does not abolish viroid infectivity in eggplants^20^. As an RNA of interest, we chose the RNA aptamer Spinach^13^, a 98-nt-long RNA that emits green fluorescence, which is comparable in brightness to that of fluorescent proteins, when binding to the 3′5-difluoro-4-hydroxybenzylidene (DFHBI) fluorophore. We constructed plasmid pLELVd-Spinach (Supplementary Fig. S14) in which a cDNA coding for Spinach was inserted between positions T245-T246 of the cDNA coding for ELVd. *E. coli* BL21(DE3) was co-transformed with pLELVd-Spinach and p15tRnlSm. A recombinant clone was grown and the tRNA ligase expression was induced. Aliquots of the culture were taken at different time points after induction. Interestingly, chimeric ELVd-Spinach RNA also accumulated in the recombinant *E. coli* cells in vast amounts (Fig. 4a, lane 7). We estimated an accumulation of approximately 75 mg of circular and linear ELVd-Spinach RNA per liter of bacterial culture at 12 h post-induction (Supplementary Fig. S15). The circular:linear ratio was again 3:1. We observed intense green fluorescence when adding DFHBI to either an aliquot of intact cells or an RNA preparation, which demonstrated that a properly folded Spinach aptamer was produced (Fig. 4b). Next, we analyzed how the partial deletion of the ELVd moiety affected the production of the RNA of interest. We chose the deleted form ELVd L3R1 that conserved the U245-U246 insertion site, and prepared plasmid pLELVdL3R1-Spinach, in which the Spinach cDNA was inserted into the deleted viroid cDNA (Supplementary Fig. S14). Despite the production of the corresponding chimeric RNA being lower than that achieved with full-length ELVd, it still obtained the notable amount of 30 mg of circular and linear ELVd L3R1-Spinach RNA per liter of bacterial culture (Supplementary Fig. S16). In this particular experiment circular:linear ratio was 9:1. We also tested whether the chimeric ELVd-Spinach RNA was infectious in eggplants. Results were negative, in contrast to what occurred with the wild-type ELVd control (Supplementary Fig. S17).

**Figure 5:**
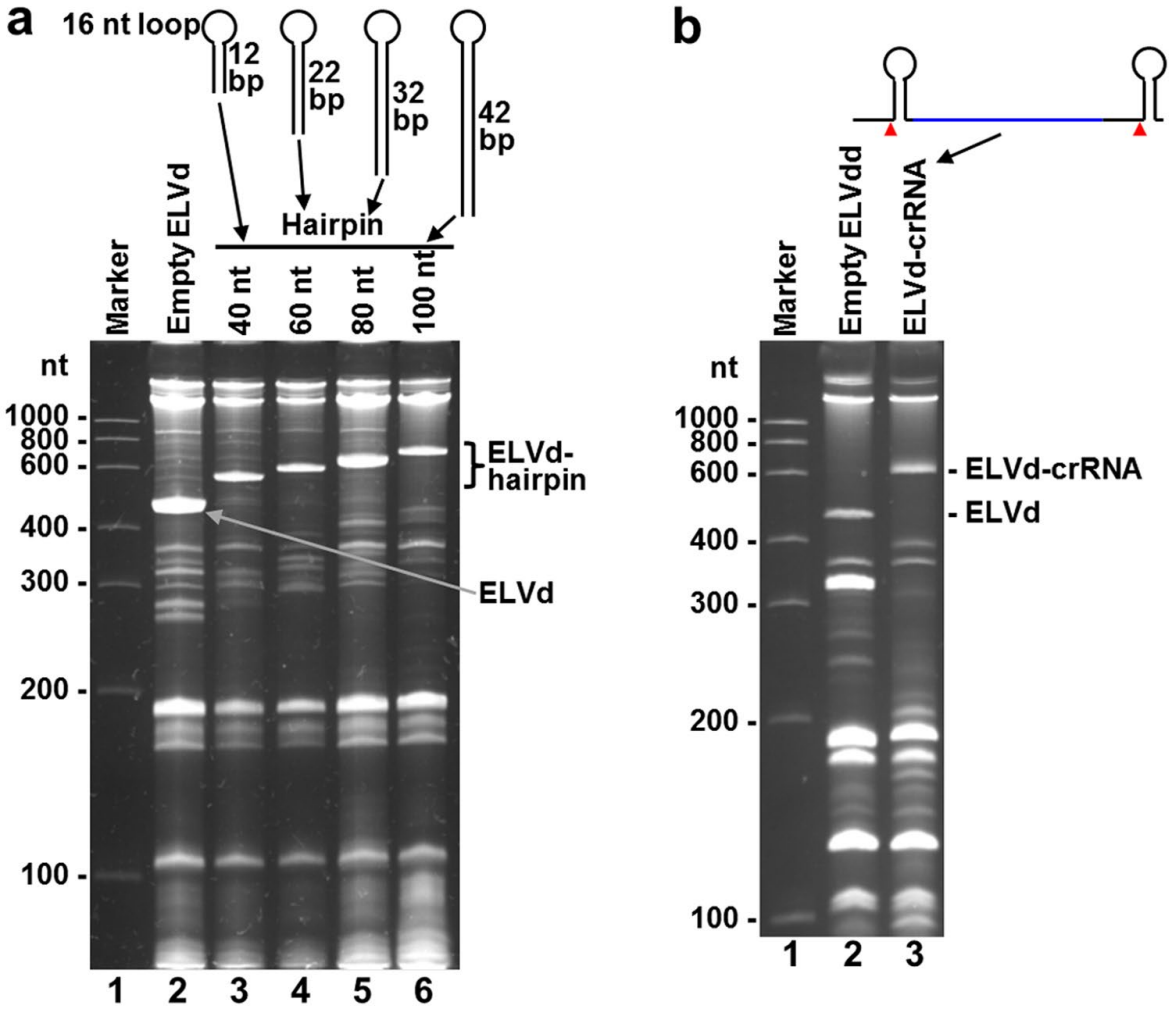
Production of extended hairpins and a crRNA in *E. coli* using the viroid-derived system. RNAs were extracted from the aliquots of *E. coli cultures* taken at 15.5 h post-inoculation, separated by denaturing PAGE, and gels were stained with ethidium bromide. (**a**) Hairpin RNAs were produced in the RNase III-deficient *E. coli* strain HT115(DE3) co-transformed with p15LtRnlSm and the different pLELVd-hairpin. Lane 1, RNA marker with the sizes of the standards indicated on the left in nt; lanes 2 to 6, RNAs from the *E. coli* transformed to express empty ELVd (lane 2) and the different ELVd forms that included hairpin RNAs of 40, 60, 80 and 100 nt, as indicated (lanes 3 to 6). The positions of the circular empty ELVd and the different chimeric ELVd-hairpin RNAs are indicated on the right. *E. coli* cultures were grown at 37 °C in TB medium. Each lane contains an aliquot of RNA that corresponds to 0.4 ml of culture. (**b**) crRNA was produced in *E. coli* BL21(DE3) co-transformed with p15LtRnlSm and pLELVd-crRNA. Lane 1, RNA marker with the sizes of the standards indicated on the left in nt; lanes 2 and 3, RNAs from the *E. coli* transformed to express empty ELVd and the chimeric ELVd-crRNA, respectively. The positions of the circular empty ELVd and the chimeric ELVd-crRNA are indicated on the right. *E. coli* cultures were grown at 37 °C in TB medium and cells harvested at 15.5 h post-inoculation. Each lane contains an aliquot of RNA that corresponds to 0.8 ml of culture.

We wondered whether this system would also serve to produce recombinant RNAs with extended double-stranded motifs, like those that induce RNA silencing^14^. To answer this question, we constructed plasmids to express RNA hairpin structures of different lengths, which ranged from 40 to 100 nt (pLELVd-hairpin40, pLELVd-hairpin60, pLELVd-hairpin80 and pLELVd-hairpin100; Supplementary Fig. S18). Initial attempts to overproduce these chimeric RNAs in *E. coli* BL21(DE3) failed (Supplementary Fig. S19), but hairpin RNAs were efficiently produced (Fig. 5a) when we used the *E. coli* strain HT115(DE3) that lacked RNase III^21^. Finally, we also produced a 65-nt-long structured RNA moiety consisting of the precursor of a crRNA with two repeated hairpins, like those that guide a Cpf1 nuclease for genome editing^15^ (Fig. 5b and Supplementary Fig. S20).

## Discussion

Unlike other central players of biological processes like DNA or proteins, the large amounts of recombinant RNA molecules required for research and biotechnological applications are not easily produced *in vitro* or *in vivo*, possibly due to the intrinsically short half-life of RNA. However in recent years, some successful strategies, based mainly on using very stable RNA scaffolds^22^ or on producing stable ribonucleoprotein complexes^23^, have been developed to produce recombinant RNA in *E. coli* cultures. We herein present a new system that incorporates both concepts and which, to our understanding, produces much larger amounts of recombinant RNAs than those previously reported. The viroid molecule is an extremely stable RNA scaffold. Indeed, viroids have been shaped by evolution to survive as naked RNAs in the hostile milieu of infected plant cells. The co-expressed eggplant tRNA ligase recognizes the ELVd moiety of recombinant RNA, and most probably participates in the formation of a stable ribonucleoprotein complex that accumulates in vast amounts in *E. coli* cells. This large accumulation occurs despite the absence of viroid replication intermediates supports no ELVd replication in *E. coli*. tRNA ligase activity also contributes to the production of recombinant RNAs as circular forms, which must exhibit further stability *in vivo* due to resistance to exonuclease attack. However, our experimental results also indicate that ELVd and derived RNAs are finally degraded in *E. coli* at late culture phases and an optimum time must be determined to harvest the recombinant RNA. Another advantage of circularity is that the absence of endogenous circular RNAs in *E. coli* allows the easy purification of recombinant RNA to virtual homogeneity (Supplementary Fig. S21). It is true that when using this system recombinant RNAs are produced as chimeric molecules, which include a viroid moiety. Although we also show that the viroid component can be partially reduced, methods have been described to excise RNA of interest from these kinds of hybrid molecules^24^. Concerns about our methodology may arise from using an infectious agent like ELVd. However, ELVd has never been transmitted to other plants, apart from its natural host, the eggplant, where it induces latent infections with no apparent symptoms^12^. Moreover, ELVd chimeric forms with inserted RNAs of interest and, obviously, those that included extended viroid deletions, were not infectious at all.

Viroids are a class of infectious agents of higher plants that exclusively consist of small non-protein-coding circular RNA molecules that occupy the bottom step in the scale of life^25^. Despite this simplicity, they are able to direct their own replication in infected cells and movement through infected plants, frequently inducing disease^26^. Here we show that, like viruses, they can also be transformed into useful biotechnological tools to produce large amounts of recombinant RNAs in bacterial cells, such as aptamers or other structured RNAs. Most importantly, by using the viroid-derived system we accomplished the amazing production of 75 mg of ELVd-Spinach RNA per liter of bacterial culture under standard laboratory conditions.

## Methods

### Plasmid construction

All plasmids were constructed using standard molecular cloning techniques. DNAs were amplified by the polymerase chain reaction (PCR) using the Phusion High-Fidelity DNA polymerase (Thermo Scientific). DNAs were digested with the type-IIS restriction enzyme *Bpi* I (Thermo Scientific) and assembled^27^ into plasmid by ligation with T4 DNA ligase (Thermo Scientific). The sequences of the resulting plasmids were experimentally confirmed (3130xl Genetic Analyzer, Life Technologies).

### *Escherichia coli* culture

The strains BL21(DE3) (Novagen) and HT115(DE3)^21^ of *E. coli* were electroporated or co-electroporated (ECM 399, BTX) with the different plasmids and recombinant clones selected at 37 °C in plates of LB solid medium (10 g/l tryptone, 5 g/l yeast extract, 10 g/l NaCl and 1.5% agar) that included the appropriate antibiotics (50 µg/ml ampicillin, 34 µg/ml chloramphenicol or both). *E. coli* was grown in liquid cultures in LB or TB medium (12 g/l tryptone, 24 g/l yeast extract, 0.4% glycerol, 0.17 M KH_2_PO_4_ and 0.72 M K_2_HPO_4_), containing the appropriate antibiotics (see above), at the indicated temperature (in general 37 °C) with vigorous shaking (225 revolutions per min –rpm–). Cell densities were measured at 600 nm with a colorimeter (CO8000, WPA). When needed, induction of eggplant tRNA ligase expression was carried out by adding the appropriate amount of 0.25 M IPTG to the culture.

### RNA extraction and analysis

At the desired time points, 2-ml aliquots of the liquid cultures were taken and cells were sedimented by centrifuging at 13,000 rpm for 2 min. In general, cells were resuspended in 50 µl of TE buffer (10 mM Tris-HCl, pH 8.0 and 1 mM EDTA) by vortexing (40-fold concentration). One volume (50 µl) of a 1:1 (v/v) mix of phenol (saturated with water and equilibrated at pH 8.0 with Tris-HCl, pH 8.0) and chloroform was added and the cells broken by vigorous vortexing. The aqueous and organic phases were separated by centrifugation for 5 min at 13,000 rpm. The aqueous phases were recovered and re-extracted with one volume (50 µl) of chloroform. The aqueous phases containing total bacterial nucleic acids were finally recovered by pipetting and either subjected directly to further analyses or stored frozen at −20 °C.

Total RNA from *E. coli* was analyzed by denaturing PAGE. In general, 20 µl of RNA preparations were mixed with 1 volume (20 µl) of loading buffer (98% formamide, 10 mM Tris-HCl, pH 8.0, 1 mM EDTA, 0.0025% bromophenol blue and 0.0025% xylene cyanol), heated for 1.5 min at 95 °C and snap cooled on ice. Electrophoresis was run for 2 h and 30 min at 200 V in 5% polyacrylamide gels (37.5:1 acrylamyde:*N,N’*-methylenebisacrylamide) of 140 × 130 × 2 mm in TBE buffer (89 mM Tris, 89 mM boric acid, 2 mM EDTA) including 8 M urea. Electrophoresis buffer was TBE without urea. Gels were stained by shaking for 15 min in 200 ml of 1 µg/ml ethidium bromide. After washing three times with water, gels were photographed under UV light (UVIdoc-HD2/20MX, UVITEC Cambridge). Image analyses were performed using the UVIDoc 1D software (UVITEC Cambridge). In some experiments (e.g. Fig. 2c and d), RNAs separated by single denaturing PAGE in TBE buffer were subjected to a second denaturing electrophoresis in lower (0.25 × TBE) ionic strength. After the first dimension, the whole lane from 5% polyacrylamide, 8 M urea, TBE gel was cut and laid transversally on top of a 5% polyacrylamide, 8 M urea, 0.25 × TBE gel of the same dimensions and run for 2.5 h at 350 V. An upper limit of 25 mA was set.

For northern blot hybridization analysis, RNAs separated by electrophoresis were electroblotted to positively charged nylon membranes (Nytran SPC, Whatman) and cross-linked by irradiation with 1.2 J/cm^2^ UV light (254 nm, Vilber Lourmat). Hybridization was performed overnight at 70 °C in 50% formamide, 0.1% Ficoll, 0.1% polyvinylpyrrolidone, 100 ng/ml salmon sperm DNA, 1% sodium dodecyl sulfate –SDS–, 0.75 M NaCl, 75 mM sodium citrate, pH 7.0, with approximately 1 million counts per minute of ^32^P-labelled ELVd RNA of complementary polarity. Hybridized membranes were washed three times for 10 min with 2 × SSC, 0.1% SDS at room temperature and once for 15 min at 55 °C with 0.1 × SSC, 0.1% SDS (1 × SSC is 150 mM NaCl, 15 mM sodium citrate, pH 7.0). Results were registered by autoradiography using X-ray films (Fujifilm). The radioactive probes were produced by *in vitro* transcription of a linearized plasmids containing a dimeric ELVd (sequence variant AJ536613) cDNA in the proper orientation. One µg of linearized plasmid (*Hin*d III or *Xba* I) was transcribed with 50 U of T3 bacteriophage RNA polymerase (Epicentre) in a 20-µl reaction containing 40 mM Tris-HCl, pH 8.0, 6 mM MgCl_2_, 20 mM DTT, 2 mM spermidine, 0.5 mM each of ATP, CTP, and GTP, and 50 μCi of [α-^32^P]UTP (800 Ci/mmol), 20 U RNase inhibitor (RiboLock, Thermo Scientific) and 0.1 U yeast inorganic pyrophosphatase (Thermo Scientific). Reactions were incubated for 1 h at 37 °C. After transcription, the DNA template was digested with 20 U DNase I (Thermo Scientific) for 10 min at 37 °C, and the probes were purified by chromatography using a Sephadex G-50 column (Mini Quick Spin Column, Roche Applied Science).

### ELVd RNA purification

For preparative purposes (Supplementary Fig. S21), 1 l of total *E. coli* culture in TB medium distributed in four 1 l baffled Erlenmeyer flasks was grown at 37 °C with intense shaking (180 rpm). Protein expression was induced at an OD_600_ of 0.1 by adding IPTG to 0.1 mM. Cells were recovered at 14 h post-induction by centrifugation at 10,000 rpm for 10 min. Sedimented cells were resuspended in water and pelleted again in the same conditions. Cells were resuspended in 50 ml of chromatography buffer (50 mM Tris-HCl, pH 6.5, 0.15 M NaCl, 0.2 mM EDTA) and RNA extracted by adding 50 ml phenol:chloroform (1:1, pH 8.0) and vortexing. The aqueous phase, recovered after centrifugation for 10 min at 10,000 rpm, was re-extracted with 50 ml of chloroform. RNA in the second aqueous phase was filtered (0.2 µm, Filtropur S, Sarstedt) and chromatographed through a DEAE Sepharose column (HiTrap DEAE FF, GE Healthcare) of 5 ml at 5 ml/min using an ÄKTA Prime Plus liquid chromatography system (GE Healthcare). The column was equilibrated with 50 ml of chromatography buffer and the sample (35 ml at this point) loaded. The column was washed with 50 ml of chromatography buffer and the RNA eluted with 100 ml of chromatography buffer plus 1 M NaCl. Fractions (5 ml) were collected during elution and analyzed by denaturing PAGE. Chromatography fraction 2, corresponding to the RNA elution peak, was mixed with 1 volume of formamide loading buffer and the RNA denatured (see above). RNA was separated by two consecutive electrophoreses. First by PAGE in a 5% polyacrylamide, 8 M urea, TBE gel, as described above. After staining with ethidium bromide, the band of the gel containing the monomeric circular ELVd RNA was cut and loaded on top of a second non-denaturing gel (5% polyacrylamide −39:1 acrylamyde:N,N’-bis(acryloyl)cystamine–, TAE −40 mM Tris, 20 mM sodium acetate, 1 mM EDTA, pH 7.2–). After staining, the band of gel containing the monomeric circular ELVd RNA was cut and solubilized by adding 0.1 volumes of 2-mercaptoethanol. RNA was purified from the solution by chromatography with a DEAE Sepharose column as explained above.

### Fluorescence assay

To assay the fluorescence of RNA aptamer Spinach, aliquots of the *E. coli* cultures were supplemented with 200 µM DFHBI and grown for 1 additional h. Pelleted bacteria were photographed under a stereomicroscope (Leica MZ 16 F) with UV illumination and a GFP2 filter (Leica). In the case of the RNA extracts, DFHBI was directly added to 20 µM and photographed under the same conditions.

## Electronic supplementary material

Supplementary Information
