# Supplementary material for: A viroid-derived system to produce large amounts of recombinant RNA in Escherichia coli
Source: Sci Rep. 2018 Jan 30;8:1904. doi: 10.1038/s41598-018-20314-3 (PMC5789856; doi:10.1038/s41598-018-20314-3)
Supplement: Supplementary file 1 — Supplementary Information [file 41598_2018_20314_MOESM1_ESM.pdf]

SUPPLEMENTARY INFORMATION

A viroid-derived system to produce large amounts of recombinant RNA in *Escherichia coli*

José-Antonio Daròs*, Verónica Aragonés & Teresa Cordero

Instituto de Biología Molecular y Celular de Plantas (Consejo Superior de Investigaciones Científicas-Universitat Politècnica de València), 46022 Valencia, Spain. Correspondence and requests for materials should be addressed to J.-A.D. (email: jadaros@ibmcp.upv.es)

Figure S1. Nucleotide sequences and elements of plasmids pLELVd, p15tRnlSm and p15mCherry.

>pLELVd (2050 bp)
CGATGCTTCTTTGAGCGAACGATCAAAAATAAGTGCCTTCCCATCAAAAAAATATTCTCAACATAAAAAACTTTG
TGTAATACTTGTAACGCTGCCCCATAGGGTGGTGTGTGCCACCCCTGATGAGACCGAAAGGTCGAAATGGGGTTT
CGCCATGGGTCGGGACTTTAAATTCGGAGGATTCGTCCTTTAAACGTTCCTCCAAGAGTCCCTTCCCCAAACCCT
TACTTTGTAAGTGTGGTTCGGCGAATGTACCGTTTCGTCCTTTCGGACTCATCAGGGAAAGTACACACTTTCCGA
CGGTGGGTTCGTCGACACCTCTCCCCCTCCCAGGTACTATCCCCTTTCAAGGATGTGTTCCCTAGGAGGGTGGGT
GTACCTCTTTTGGATTGCTCCGGCCTTCCAGGAGAGATAGAGGACGACCTCTCCCCATAGGGTGGTGTGTGCCAC
CCCTGATGAGACCGAAAGGTCGAAATGGGGGAAATCATCCTTAGCGAAAGCTAAGGATTTTTTTTATCTGAAATG
CGTTGCTGGCGTTTTTCCATAGGCTCCGCCCCCCTGACGAGCATCACAAAAATCGACGCTCAAGTCAGAGGTGGC
GAAACCCGACAGGACTATAAAGATACCAGGCGTTTCCCCCTGGAAGCTCCCTCGTGCGCTCTCCTGTTCCGACCC
TGCCGCTTACCGGATACCTGTCCGCCTTTCTCCCTTCGGGAAGCGTGGCGCTTTCTCATAGCTCACGCTGTAGGT
ATCTCAGTTCGGTGTAGGTCGTTCGCTCCAAGCTGGGCTGTGTGCACGAACCCCCCGTTCAGCCCGACCGCTGCG
CCTTATCCGGTAACTATCGTCTTGAGTCCAACCCGGTAAGACACGACTTATCGCCACTGGCAGCAGCCACTGGTA
ACAGGATTAGCAGAGCGAGGTATGTAGGCGGTGCTACAGAGTTCTTGAAGTGGTGGCCTAACTACGGCTACACTA
GAAGGACAGTATTTGGTATCTGCGCTCTGCTGAAGCCAGTTACCTTCGGAAAAAGAGTTGGTAGCTCTTGATCCG
GCAAACAAACCACCGCTGGTAGCGGTGGTTTTTTTGTTTGCAAGCAGCAGATTACGCGCAGAAAAAAAGGATCTC
AAGAAGATCCTTTTTACCAATGCTTAATCAGTGAGGCACCTATCTCAGCGATCTGTCTATTTCGTTCATCCATAG
TTGCCTGACTCCCCGTCGTGTAGATAACTACGATACGGGAGGGCTTACCATCTGGCCCCAGTGCTGCAATGATAC
CGCGAGAGCCACGCTCACCGGCTCCAGATTTATCAGCAATAAACCAGCCAGCCGGAAGGGCCGAGCGCAGAAGTG
GTCCTGCAACTTTATCCGCCTCCATCCAGTCTATTAATTGTTGCCGGGAAGCTAGAGTAAGTAGTTCGCCAGTTA
ATAGTTTGCGCAACGTTGTTGCCATTGCTACAGGCATCGTGGTGTCACGCTCGTCGTTTGGTATGGCTTCATTCA
GCTCCGGTTCCCAACGATCAAGGCGAGTTACATGATCCCCCATGTTGTGCAAAAAAGCGGTTAGCTCCTTCGGTC
CTCCGATCGTTGTCAGAAGTAAGTTGGCCGCAGTGTTATCACTCATGGTTATGGCAGCACTGCATAATTCTCTTA
CTGTCATGCCATCCGTAAGATGCTTTTCTGTGACTGGTGAGTACTCAACCAAGTCATTCTGAGAATAGTGTATGC
GGCGACCGAGTTGCTCTTGCCCGGCGTCAATACGGGATAATACCGCGCCACATAGCAGAACTTTAAAAGTGCTCA
TCATTGGAAAACGTTCTTCGGGGCGAAAACTCTCAAGGATCTTACCGCTGTTGAGATCCAGTTCGATGTAACCCA
CTCGTGCACCCAACTGATCTTCAGCATCTTTTACTTTCACCAGCGTTTCTGGGTGAGCAAAAACAGGAAGGCAAA
ATGCCGCAAAAAAGGGAATAAGGGCGACACGGAAATGTTGAATACTCATACTCTTCCTTTTTCAATATTATTGAA
GCATTTATCAGGGTTATTGTCTCAT

E. coli **murein lipoprotein promoter** is in red and **ELVd** cDNA **(**C327 to G46 of AJ536613) is in black with the repeated hammerhead ribozyme domain on yellow background. Ribozymes self-cleavage sites are underlined. *E. coli* ribosomal **rrnC terminator** is in fuchsia. **pUC replication origin** is in gray and **ampicillin resistance gene** (inverse orientation) on gray background (**promoter** on dark gray background).

>p15tRnlSm (5338 bp)
TAATACGACTCACTATAGGGAGACCACAACGGTTTCCCTCTAGAAATAATTTTGTTTAACTTTAAGAAGGAGATA
TACCATGTCGGTTTCGCATAGGGTCATTTACTCTTTCACTCATTACAAACTCTATAATCTCTCTTCTTCTTTATC
ATCTTTGCCTTCTAGAATCTTCTTCCCTTTTCAATCTCCTTCCTTTCACACGTTCTCTTCACTCATGCCCAACAA
TCAGGAAAGGGGTGGTTATGAAGGAAAAAAATGGCAAGTGAGGCCAAGTTCCAATAGGGTACCAGGCTCGTCTTC
AAATGTGGAACCTGTATCTGCTGCAACTGCTGAAGCCATTACCGACCGTCTAAAGTCCGTGGATATTACTGAAAG
TGGTGCACAGTCTAGTGTTCCAGTCACATCTCTTCAGTTTGGCAGCGTTGGATTAGCACCCCAGTCACCTGTGCA
ACATCAAAAAGTAATCTGGAAACCCAAATCATATGGAACAGTGTCTGGAGCCCCAGTGGTTGAAGCTGGAAAAAC
ACCAGTTGAACAAAAAAGTGCTCTTTTAAGTAAATTATTCAAGGGTAATTTATTGGAAAATTTTACTGTAGATAA
CTCAACATTCTCGAGAGCCCAAGTAAGGGCCACTTTCTACCCAAAATTTGAGAATGAGAAATCAGATCAGGAGAT
CAGGACAAGGATGATAGAGATGGTCTCCAAAGGCTTGGCTATAGTCGAGGTCACACTTAAGCATTCTGGATCTCT
TTTTATGTATGCTGGGCATGAAGGTGGAGCATATGCCAAGAATAGCTTCGGGAATATCTATACTGCCGTTGGCGT
CTTTGTTCTTGGACGGATGTTTCGTGAGGCATGGGGAACTAAAGCAAGCAAGAAGCAAGCAGAGTTCAATGAGTT
TCTTGAGCGCAATCGTATGTGCATATCAATGGAGTTGGTCACGGCAGTGTTGGGGGACCACGGACAACGCCCACG
AGATGATTATGCGGTTGTGACTGCAGTCACGGAGTTGGGAAATGGAAAACCAACTTTCTATTCAACTCCCGATGT
AATTGCTTTTTGCAGGGAATGGCGATTACCAACAAATCATGTATGGCTGTTCTCAACAAGGAAATCAGTGACTTC
CTTCTTTGCTGCGTATGATGCACTTTGCGAGGAAGGTACAGCAACCACCGTTTGCGAGGCTCTCAGCGAAGTTGC
TGATATTTCTGTACCTGGATCAAAAGACCATATAAAAGTGCAGGGTGAAATTTTGGAGGGTCTCGTGGCCCGCAT
CGTAAAACGTGAGAGCTCAGAGCATATGGAGCGGGTTCTGAGAGATTTTCCTCCTCCGCCATCAGAGGGTGAGGG
TTTGGACCTGGGACCTACGTTACGTGAAATTTGTGCTGCAAACAGATCAGAAAAGCAGCAAATAAAGGCACTTCT

TCAGAGTGCTGGCACGGCTTTCTGCCCGAATTATTTGGACTGGTTTGGAGATGAAAACTCTGGTTCACATTCAAG
AAATGCTGATCGATCTGTTGTCTCAAAGTTCTTACAATCACATCCTGCTGATCTTTATACAGGAAAAATACAGGA
AATGGTTCGCTTGATGAGGGAAAAGCGCTTTCCTGCTGCTTTCAAGTGTCATTATAACTTACATAAAATTAATGA
TGTATCGAGTAACAACCTGCCTTTCAAAATGGTGATCCATGTATATAGTGATTCAGGCTTCCGCCGGTACCAGAA
AGAGATGAGGCACAAACCAGGACTATGGCCTTTGTATCGAGGCTTTTTTGTTGACCTGGATTTATTCAAGGTCAA
TGAGAAGAAAACTGCTGAAATGGCAGGAAGCAACAATCAAATGGTAAAAAATGTGGAAGAGGACAACAGTTTAGC
TGATGAAGATGCAAATCTGATGGTCAAGATGAAATTTCTTACTTACAAGTTGAGAACTTTTTTGATCCGTAATGG
CTTGTCGACTCTTTTCAAAGAAGGACCTTCTGCGTATAAGTCTTATTACCTGAGGCAAATGAAAATTTGGAATAC
TTCAGCAGCCAAGCAACGAGAACTCAGCAAGATGCTTGATGAATGGGCAGTATATATACGCAGAAAATATGGGAA
CAAACCATTGTCATCATCCACATACCTAAGTGAAGCTGAGCCTTTCCTTGAACAATATGCAAAGCGTAGTCCACA
AAATCATGCTTTGATAGGATCTGCTGGAAATTTTGTCAAAGTTGAAGATTTCATGGCTATTGTTGAAGGAGAAGA
TGAAGAGGGTGATCTCGAGCCTGCGAAAGATATTGCTCCTTCAAGCCCTAGTATTTCCACCAGAGACATGGTGGC
AAAGAATGAGGGTCTCATTATTTTCTTTCCAGGAATACCAGGTTGTGCTAAATCTGCACTTTGTAAGGAAATACT
GAATGCTCCAGGAGGGCTTGGAGATGATCGACCAGTTAACAGTTTAATGGGTGATCTTATTAAAGGTAGATATTG
GCAAAAAGTTGCTGATGAACGTCGAAGAAAACCTTACTCGATCATGCTTGCTGACAAGAATGCACCAAATGAGGA
AGTATGGAAACAAATTGAGAACATGTGCCTAAGCACCGGAGCATCTGCTATTCCAGTTATACCTGATTCAGAAGG
AACTGAAACTAATCCATTCTCTATTGATGCACTTGCGGTTTTTATATTCCGAGTACTTCACCGTGTCAATCATCC
GGGAAATCTTGACAAGTCATCTCCAAATGCTGGATATGTGATGCTTATGTTTTATCACCTTTATGATGGAAAGAG
CCGTCAGGAGTTCGAGAGTGAGCTTATTGAACGTTTTGGATCGCTTGTCAGAATTCCTGTACTGAAACCTGAGAG
GTCTCCTCTTCCGGATTCTGTGAGGTCTATTATCGAGGAGGGACTCAGTCTGTACAGACTTCATACAACGAAACA
TGGAAGATTGGAGTCTACAAAAGGGACATATGTACAAGAGTGGGTTAAATGGGAGAAGCAATTGAGAGATATTCT
ACTTGGAAATGCAGACTATCTCAATTCAATACAGGTTCCATTTGAATTTGCCGTTAAAGAAGTCCTTGAACAACT
GAAAGTTATTGCGAGGGGCGAATATGCAGTGCCTGCTGAGAAGAGGAAGCTAGGATCCATTGTATTCGCCGCTAT
CAGCCTGCCAGTTCCAGAAATTCTAGGTCTTCTAAATGATCTAGCAAAGAAAGATCCAAAGGTTGGCGATTTCAT
TAAGGACAAGAGCATGGAGAGCAGCATTCAGAAGGCCCATCTTACCCTGGCTCACAAGAGAAGTCACGGTGTCAC
TGCAGTTGCCAATTACGGTTCCTTTCTTCATCAAAAGGTGCCAGTAGACGTGGCTGCTTTGTTGTTCTCCGATAA
ATTGGCTGCACTAGAAGCTGAGCCTGGCTCTGTTGAAGGTGAAAAGATCAATTCTAAAAACTCATGGCCCCATAT
CACATTATGGTCTGGTGCAGGAGTTGCCGCAAAAGATGCCAATACACTACCACAGTTACTTTCCCAAGGGAAGGC
TACCCGCATTGATATAAATCCACCGGTCACTATAACTGGCACTCTCGAATTCTTTCACCACCACCACCACCACTG
AGATCCGGCTGCTAACAAAGCCCGAAAGGAAGCTGAGTTGGCTGCTGCCACCGCTGAGCAATAACTAGCATAACC
CCTTGGGGCCTCTAAACGGGTCTTGAGGGGTTTTTTGCTGAAAGGAGATCTGGCGGGGCCCGCGCTAGCGGAGTG
TATACTGGCTTACTATGTTGGCACTGATGAGGGTGTCAGTGAAGTGCTTCATGTGGCAGGAGAAAAAAGGCTGCA
CCGGTGCGTCAGCAGAATATGTGATACAGGATATATTCCGCTTCCTCGCTCACTGACTCGCTACGCTCGGTCGTT
CGACTGCGGCGAGCGGAAATGGCTTACGAACGGGGCGGAGATTTCCTGGAAGATGCCAGGAAGATACTTAACAGG
GAAGTGAGAGGGCCGCGGCAAAGCCGTTTTTCCATAGGCTCCGCCCCCCTGACAAGCATCACGAAATCTGACGCT
CAAATCAGTGGTGGCGAAACCCGACAGGACTATAAAGATACCAGGCGTTTCCCCCTGGCGGCTCCCTCGTGCGCT
CTCCTGTTCCTGCCTTTCGGTTTACCGGTGTCATTCCGCTGTTATGGCCGCGTTTGTCTCATTCCACGCCTGACA
CTCAGTTCCGGGTAGGCAGTTCGCTCCAAGCTGGACTGTATGCACGAACCCCCCGTTCAGTCCGACCGCTGCGCC
TTATCCGGTAACTATCGTCTTGAGTCCAACCCGGAAAGACATGCAAAAGCACCACTGGCAGCAGCCACTGGTAAT
TGATTTAGAGGAGTTAGTCTTGAAGTCATGCGCCGGTTAAGGCTAAACTGAAAGGACAAGTTTTGGTGACTGCGC
TCCTCCAAGCCAGTTACCTCGGTTCAAAGAGTTGGTAGCTCAGAGAACCTTCGAAAAACCGCCCTGCAAGGCGGT
TTTTTCGTTTTCAGAGCAAGAGATTACGCGCAGACCAAAACGATCTCAAGAAGATCATCTTATTAATTACGCCCC
GCCCTGCCACTCATCGCAGTACTGTTGTAATTCATTAAGCATTCTGCCGACATGGAAGCCATCACAGACGGCATG
ATGAACCTGAATCGCCAGCGGCATCAGCACCTTGTCGCCTTGCGTATAATATTTGCCCATGGTGAAAACGGGGGC
GAAGAAGTTGTCCATATTGGCCACGTTTAAATCAAAACTGGTGAAACTCACCCAGGGATTGGCTGAGACGAAAAA
CATATTCTCAATAAACCCTTTAGGGAAATAGGCCAGGTTTTCACCGTAACACGCCACATCTTGCGAATATATGTG
TAGAAACTGCCGGAAATCGTCGTGGTATTCACTCCAGAGCGATGAAAACGTTTCAGTTTGCTCATGGAAAACGGT
GTAACAAGGGTGAACACTATCCCATATCACCAGCTCACCGTCTTTCATTGCCATACGGAATTCCGGATGAGCATT
CATCAGGCGGGCAAGAATGTGAATAAAGGCCGGATAAAACTTGTGCTTATTTTTCTTTACGGTCTTTAAAAAGGC
CGTAATATCCAGCTGAACGGTCTGGTTATAGGTACATTGAGCAACTGACTGAAATGCCTCAAAATGTTCTTTACG
ATGCCATTGGGATATATCAACGGTGGTATATCCAGTGATTTTTTTCTCCAT*TTTAGCTTCCTTAGCTCCTGAAAA*
*TCTCGATAACTCAAAAAATACGCCCGGTAGTGATCTTATTTCATTATGGTGAAAGTTGGAA*CCCGGGGGCGGCGG
CCGCCCGCGAAAT

T7 bacteriophage promoter underlined with the **+1** nucleotide of the transcript on yellow background. Eggplant tRNA ligase (JX0225157) in black with the theoretical amino-terminal **transit peptide** on green background and the carboxy-terminal **hexahistidine tag** in blue. **Stop codon** in red. **T7 bacteriophage terminator** on red background. **p15A replication**

origin in gray and **chloramphenicol resistance gene** (reverse orientation) in blue (***promoter*** in italics).

>p15mCherry (2623 bp)

```
TAATACGACTCACTATAGGGGGACCACAACGGTTTCCCTCTAGAAATAATTTTGTTTAACTTTAAGAAGGAGATA
TACCATGGTTAGCAAAGGCGAGGAGGATAACATGGCCATCATCAAGGAGTTCATGCGCTTCAAGGTGCACATGGA
GGGCTCCGTGAACGGCCACGAGTTCGAGATCGAGGGCGAGGGCGAGGGCCGCCCCTACGAGGGCACCCAGACCGC
CAAGCTGAAGGTGACCAAGGGTGGCCCCCTGCCCTTCGCCTGGGACATCCTGTCCCCTCAGTTCATGTACGGCTC
CAAGGCCTACGTGAAGCACCCCGCCGACATCCCCGACTACTTGAAGCTGTCCTTCCCCGAGGGCTTCAAGTGGGA
GCGCGTGATGAACTTCGAGGACGGCGGCGTGGTGACCGTGACCCAGGACTCCTCCCTGCAGGACGGCGAGTTCAT
CTACAAGGTGAAGCTGCGCGGCACCAACTTCCCCTCCGACGGCCCCGTAATGCAGAAGAAGACCATGGGCTGGGA
GGCCTCCTCCGAGCGGATGTACCCCGAGGACGGCGCCCTGAAGGGCGAGATCAAGCAGAGGCTGAAGCTGAAGGA
CGGCGGCCACTACGACGCTGAGGTCAAGACCACCTACAAGGCCAAGAAGCCCGTGCAGCTGCCCGGCGCCTACAA
CGTCAACATCAAGTTGGACATCACCTCCCACAACGAGGACTACACCATCGTGGAACAGTACGAACGCGCCGAGGG
CCGCCACTCCACCGGCGGCATGGATGAGCTGTATAAGAGCGCATGGAGTCATCCTCAATTCGAGAAAGGTGGAGG
TTCTGGCGGTGGATCGGGAGGTTCAGCGTGGAGCCACCCGCAGTTCGAAAAATCCGGATGAGATCCGGCTGCTAA
CAAAGCCCGAAAGGAAGCTGAGTTGGCTGCTGCCACCGCTGAGCAATAACTAGCATAACCCCTTGGGGCCTCTAA
ACGGGTCTTGAGGGGTTTTTTGCTGAAAGGAGATCTGGCGGGGCCCGCGCTAGCGGAGTGTATACTGGCTTACTA
TGTTGGCACTGATGAGGGTGTCAGTGAAGTGCTTCATGTGGCAGGAGAAAAAAGGCTGCACCGGTGCGTCAGCAG
AATATGTGATACAGGATATATTCCGCTTCCTCGCTCACTGACTCGCTACGCTCGGTCGTTCGACTGCGGCGAGCG
GAAATGGCTTACGAACGGGGCGGAGATTTCCTGGAAGATGCCAGGAAGATACTTAACAGGGAAGTGAGAGGGCCG
CGGCAAAGCCGTTTTTCCATAGGCTCCGCCCCCCTGACAAGCATCACGAAATCTGACGCTCAAATCAGTGGTGGC
GAAACCCGACAGGACTATAAAGATACCAGGCGTTTCCCCCTGGCGGCTCCCTCGTGCGCTCTCCTGTTCCTGCCT
TTCGGTTTACCGGTGTCATTCCGCTGTTATGGCCGCGTTTGTCTCATTCCACGCCTGACACTCAGTTCCGGGTAG
GCAGTTCGCTCCAAGCTGGACTGTATGCACGAACCCCCCGTTCAGTCCGACCGCTGCGCCTTATCCGGTAACTAT
CGTCTTGAGTCCAACCCGGAAAGACATGCAAAAGCACCACTGGCAGCAGCCACTGGTAATTGATTTAGAGGAGTT
AGTCTTGAAGTCATGCGCCGGTTAAGGCTAAACTGAAAGGACAAGTTTTGGTGACTGCGCTCCTCCAAGCCAGTT
ACCTCGGTTCAAAGAGTTGGTAGCTCAGAGAACCTTCGAAAAACCGCCCTGCAAGGCGGTTTTTTCGTTTTCAGA
GCAAGAGATTACGCGCAGACCAAAACGATCTCAAGAAGATCATCTTATTAATTACGCCCCGCCCTGCCACTCATC
GCAGTACTGTTGTAATTCATTAAGCATTCTGCCGACATGGAAGCCATCACAGACGGCATGATGAACCTGAATCGC
CAGCGGCATCAGCACCTTGTCGCCTTGCGTATAATATTTGCCCATGGTGAAAACGGGGGCGAAGAAGTTGTCCAT
ATTGGCCACGTTTAAATCAAAACTGGTGAAACTCACCCAGGGATTGGCTGAGACGAAAAACATATTCTCAATAAA
CCCTTTAGGGAAATAGGCCAGGTTTTCACCGTAACACGCCACATCTTGCGAATATATGTGTAGAAACTGCCGGAA
ATCGTCGTGGTATTCACTCCAGAGCGATGAAAACGTTTCAGTTTGCTCATGGAAAACGGTGTAACAAGGGTGAAC
ACTATCCCATATCACCAGCTCACCGTCTTTCATTGCCATACGGAATTCCGGATGAGCATTCATCAGGCGGGCAAG
AATGTGAATAAAGGCCGGATAAAACTTGTGCTTATTTTTCTTTACGGTCTTTAAAAAGGCCGTAATATCCAGCTG
AACGGTCTGGTTATAGGTACATTGAGCAACTGACTGAAATGCCTCAAAATGTTCTTTACGATGCCATTGGGATAT
ATCAACGGTGGTATATCCAGTGATTTTTTTCTCCATTTTAGCTTCCTTAGCTCCTGAAAATCTCGATAACTCAAA
AAATACGCCCGGTAGTGATCTTATTTCATTATGGTGAAAGTTGGAACCCGGGGGCGGCGGCCGCCCGCGAAAT
```

T7 bacteriophage promoter underlined with the **+1** nucleotide of the transcript on yellow background. **mCherry** (AY678264) cDNA on red background, including four **silent mutations** (underlined) and a carboxy-terminal **Twin-Srep-tag** in red. **Stop codon** in red. **T7 bacteriophage terminator** on red background. **p15A replication origin** in gray and **chloramphenicol resistance gene** (reverse orientation) in blue (***promoter*** in italics).

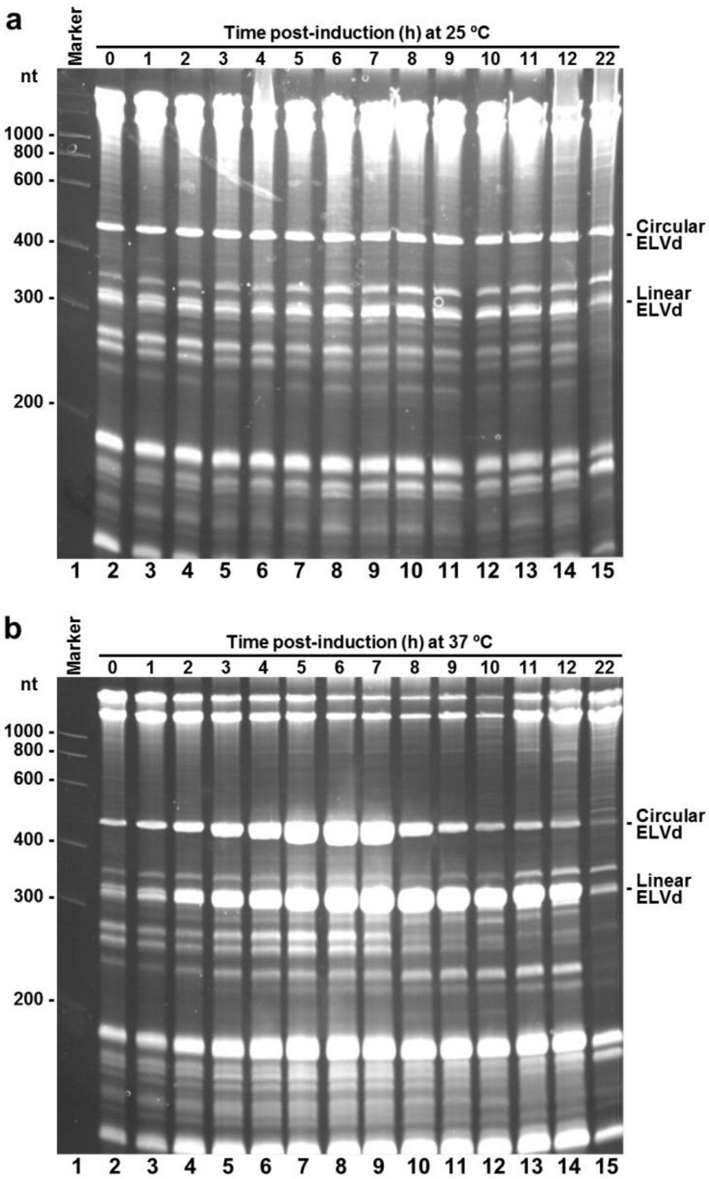

Figure S2. Time-course analysis of the RNA that accumulated in *E. coli* cells that co-expressed ELVd RNA and eggplant tRNA ligase, grown at 25ºC (a) and 37ºC (b). Aliquots of the cultures were taken at the indicated time points after induction of tRNA ligase expression. RNA was extracted and analyzed by denaturing PAGE and ethidium bromide staining. Lanes 1, RNA marker with the sizes of the standards indicated on the left in nt; lanes 2 to 15, RNA from aliquots of the cultures taken at 0, 1, 2, 3, 4, 5, 6, 7, 8, 9, 10, 11, 12 and 22 h post-induction. The positions of the circular and linear ELVd RNAs are indicated on the right. *E. coli* cultures were induced with 0.4 mM IPTG at DO_{600} 0.6. Each lane contains an aliquot of RNA corresponding to 0.8 ml of culture.

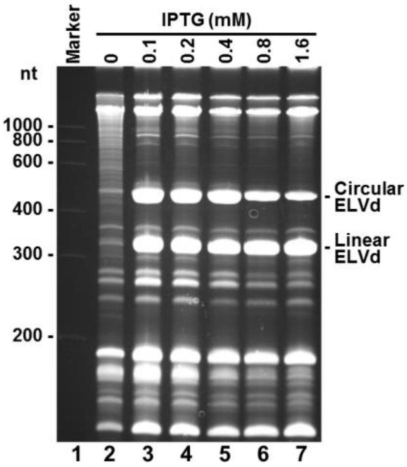

Figure S3. Analysis of the RNA that accumulated in *E. coli* cells that co-expressed ELVd RNA and eggplant tRNA ligase, induced with different amounts of IPTG. RNA preparations were separated by denaturing PAGE and the gel stained with ethidium bromide. Lane 1, RNA marker with the sizes of the standards indicated on the left in nt; lane 2, RNA from a non-induced culture; lanes 3 to 7, RNAs from cultures induced with 0.1, 0.2, 0.4, 0.8 and 1.6 mM IPTG, respectively. The positions of the circular and linear ELVd RNAs are indicated on the right. *E. coli* cultures were grown at 37ºC and induced at DO_{600} 0.6. Bacteria were harvested at 6 h post-induction. Each lane contains an aliquot of RNA corresponding to 0.8 ml of culture.

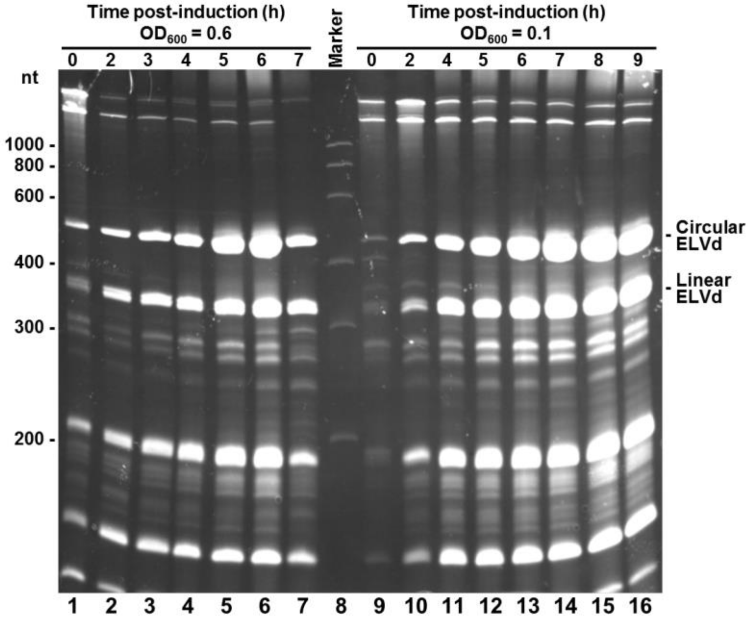

Figure S4. Time-course analysis of the RNA that accumulated in E. coli cells that co-expressed ELVd RNA and eggplant tRNA ligase, induced at different OD_{600}. Aliquots were taken from the cultures at different time points, and RNA extracted and analyzed by denaturing PAGE and ethidium bromide staining. Lanes 1 to 7, RNAs from the culture induced at OD_{600} 0.6 taken at 0, 2, 3, 4, 5, 6 and 7 h post-induction; lane 8, RNA marker with the sizes of the standards indicated on the left in nt; lanes 9 to 16, RNAs from the culture induced at OD_{600} 0.1 taken at 0, 2, 4, 5, 6, 7, 8 and 9 h post-induction. The positions of the circular and linear ELVd RNAs are indicated on the right. *E. coli* cultures were grown at 37ºC and induced with 0.1 mM ITPG. Each lane contains an aliquot of RNA corresponding to 0.4 ml of culture.

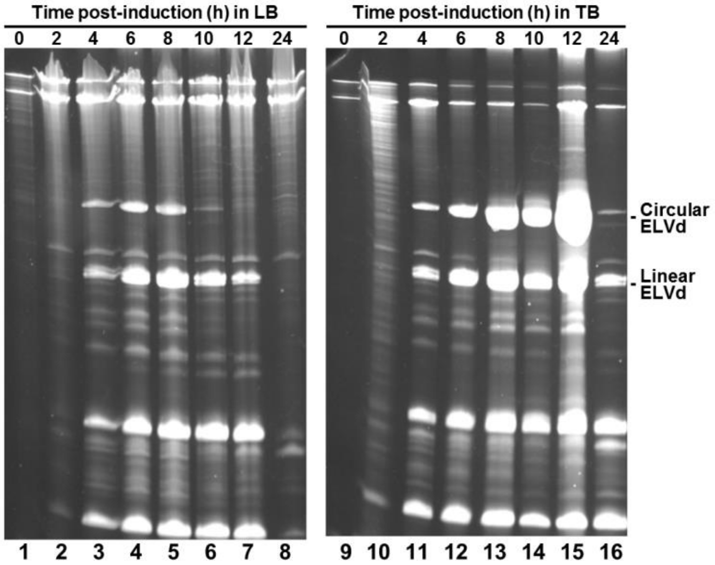

Figure S5. Time-course analysis of the RNA that accumulated in *E. coli* cells that co-expressed ELVd RNA and eggplant tRNA ligase, grown in LB and TB media. Aliquots were taken from the cultures at different time points, as indicated. RNAs were extracted and analyzed by denaturing PAGE and ethidium bromide staining of the gels. Lanes 1 to 8, RNAs from the culture grown in LB medium taken at 0, 2, 4, 6, 8, 10, 12 and 24 h post-induction; lanes 9 to 16, RNAs from the culture grown in TB medium taken at 0, 2, 4, 6, 8, 10, 12 and 24 h post-induction. The positions of the circular and linear ELVd RNAs are indicated on the right. *E. coli* cultures were grown at 37ºC and induced with 0.1 mM ITPG at DO_{600} 0.1 mM. Each lane contains an aliquot of RNA corresponding to 0.4 ml of culture.

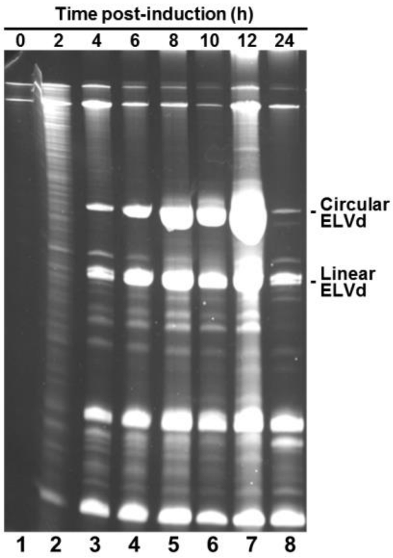

Figure S6. Time-course analysis of the RNA accumulated in *E. coli* cells that co-expressed ELVd RNA and eggplant tRNA ligase in optimized conditions (culture grown at 37ºC in TB medium and induced with 0.1 mM ITPG at DO_{600} 0.1). Aliquots from the culture were taken at different time points and RNA purified. RNAs were separated by denaturing PAGE and the gel stained with ethidium bromide. Lanes 1 to 8, RNAs from aliquots taken at 0, 2, 4, 6, 8, 10, 12 and 24 h post-induction of tRNA ligase expression. The positions of the circular and linear ELVd RNAs are indicated on the right. Each lane contains an aliquot of RNA corresponding to 0.4 ml of culture.

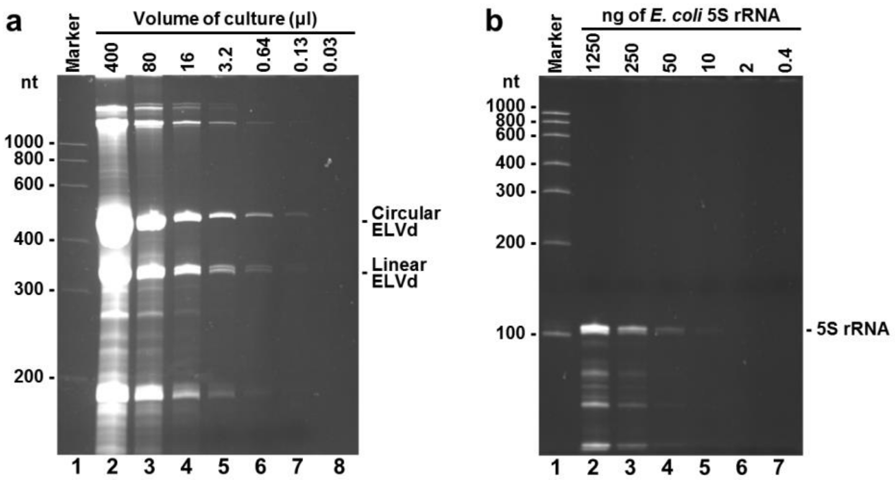

Figure S7. Quantification of the ELVd RNA produced in *E. coli* with the viroid-derived system in optimal conditions. RNA samples were separated by denaturing PAGE and the gels stained with ethidium bromide. (**a**) The RNA extracted from an aliquot of a culture taken at 12 h post-induction (see Supplementary Fig. S6) was subjected to 1/5 serial dilutions and electrophoresed. Lane 1, RNA marker with the sizes of the standards indicated on the left in nt; lanes 2 to 8, serial dilutions of the RNA preparation that corresponded to 400, 80, 16, 3.2, 0.64, 0.13 and 0.03 µl of the original culture, respectively. (**b**) A quantified preparation of *E. coli* 5S rRNA was subjected to 1/5 serial dilution before separation by PAGE and staining of the gel. Lane 1, RNA marker with the sizes of the standards indicated on the left in nt; lanes 2 to 7, 1250, 250, 50, 10, 2 and 0.4 ng of the *E. coli* 5S rRNA standard, respectively. The positions of the circular and linear ELVd RNAs, and the 5S rRNA are indicated on the right of the corresponding panel.

Figure S8. Sequences of the full-length ELVd cDNA present in plasmid pLELVd and the different ELVd deleted forms assayed in the viroid-derived system to produce recombinant RNA in *E. coli*. The position of the deletion is indicated on blue background. Hammerhead ribozyme domain is indicated on yellow background.

```
>ELVd
GGGTGGTGTGTGCCACCCCTGATGAGACCGAAAGGTCGAAATGGGGTTTCGCCATGGGTCGGACTTTAAATTCG
GAGGATTCGTCCTTTAAACGTTCCTCCAAGAGTCCCTTCCCCAAACCCTTACTTTGTAAGTGTGGTTCGGCGAAT
GTACCGTTTCGTCCTTTCGGACTCATCAGGGAAAGTACACACTTTCCGACGGTGGGTTCGTCGACACCTCTCCCC
CTCCCAGGTACTATCCCCTTTCAAGGATGTGTTCCCTAGGAGGGTGGGTGTACCTCTTTTGGATTGCTCCGGCCT
TCCAGGAGAGATAGAGGACGACCTCTCCCCATA

>ELVd L1
GGGTGGTGTGTGCCACCCCTGATGAGACCGAAAGGTCGAAATGGGGTTTCGCCATCAAACCCTTACTTTGTAAGT
GTGGTTCGGCGAATGTACCGTTTCGTCCTTTCGGACTCATCAGGGAAAGTACACACTTTCCGACGGTGGGTTCGT
CGACACCTCTCCCCCTCCCAGGTACTATCCCCTTTCAAGGATGTGTTCCCTAGGAGGGTGGGTGTACCTCTTTTG
GATTGCTCCGGCCTTCCAGGAGAGATAGAGGACGACCTCTCCCCATA

>ELVd L2
GGGTGGTGTGTGCCACCCCTGATGAGACCGAAAGGTCGAAATGGGGTTTCGCCATGGGTCGGACTTTAAATTCG
GAGGATTCGTCCTTTAAACGTTCCTCCAAGAGTCCCTTCCCCACGGCGAATGTACCGTTTCGTCCTTTCGGACTC
ATCAGGGAAAGTACACACTTTCCGACGGTGGGTTCGTCGACACCTCTCCCCCTCCCAGGTACTATCCCCTTTCAA
GGATGTGTTCCCTAGGAGGGTGGGTGTACCTCTTTTGGATTGCTCCGGCCTTCCAGGAGAGATAGAGGACGACCT
CTCCCCATA

>ELVd L3
GGGTGGTGTGTGCCACCCCTGATGAGACCGAAAGGTCGAAATGGGGTTTCGCCATTCGGCGAATGTACCGTTTCG
TCCTTTCGGACTCATCAGGGAAAGTACACACTTTCCGACGGTGGGTTCGTCGACACCTCTCCCCCTCCCAGGTAC
TATCCCCTTTCAAGGATGTGTTCCCTAGGAGGGTGGGTGTACCTCTTTTGGATTGCTCCGGCCTTCCAGGAGAGA
TAGAGGACGACCTCTCCCCATA

>ELVd L4
GGGTGGTGTGTGCCACCCCTGATGAGACCGAAAGGTCGAAATGGGGTTGTACCGTTTCGTCCTTTCGGACTCATC
AGGGAAAGTACACACTTTCCGACGGTGGGTTCGTCGACACCTCTCCCCCTCCCAGGTACTATCCCCTTTCAAGGA
TGTGTTCCCTAGGAGGGTGGGTGTACCTCTTTTGGATTGCTCCGGCCTTCCAGGAGAGATAGAGGACGACCTCTC
CCCATA

>ELVd R1
GGGTGGTGTGTGCCACCCCTGATGAGACCGAAAGGTCGAAATGGGGTTTCGCCATGGGTCGGACTTTAAATTCG
GAGGATTCGTCCTTTAAACGTTCCTCCAAGAGTCCCTTCCCCAAACCCTTACTTTGTAAGTGTGGTTCGGCGAAT
GTACCGTTTCGTCCTTTCGGACTCATCAGGGAAAGTACACACTTTCCGACGGTGGGTTCGTCGACACCTCTCCCC
CTCCCAGGTACTATCCCCTTTCAAGGATGTGTTCCCTAGGAGGGTGGGTGTACATAGAGGACGACCTCTCCCCAT
A

>ELVd R2
GGGTGGTGTGTGCCACCCCTGATGAGACCGAAAGGTCGAAATGGGGTTTCGCCATGGGTCGGACTTTAAATTCG
GAGGATTCGTCCTTTAAACGTTCCTCCAAGAGTCCCTTCCCCAAACCCTTACTTTGTAAGTGTGGTTCGGCGAAT
GTACCGTTTCGTCCTTTCGGACTCATCAGGGAAAGTACACACTTTCCGACGGTGGGTTCGTCGACCTCTTTTGGA
TTGCTCCGGCCTTCCAGGAGAGATAGAGGACGACCTCTCCCCATA

>ELVd R3
GGGTGGTGTGTGCCACCCCTGATGAGACCGAAAGGTCGAAATGGGGTTTCGCCATGGGTCGGACTTTAAATTCG
GAGGATTCGTCCTTTAAACGTTCCTCCAAGAGTCCCTTCCCCAAACCCTTACTTTGTAAGTGTGGTTCGGCGAAT
GTACCGTTTCGTCCTTTCGGACTCATCAGGGAAAGTACACACTTTCCGACGGTGGGTTCGTCGATAGAGGACGAC
CTCTCCCCATA

>ELVd R4
```

GGGTGGTGTGTGCCACCCCTGATGAGACCGAAAGGTCGAAATGGGGTTTCGCCATGGGTCGGGACTTTAAATTCG
GAGGATTCGTCCTTTAAACGTTCCTCCAAGAGTCCCTTCCCCAAACCCTTACTTTGTAAGTGTGGTTCGGCGAAT
GTACCGTTTCGTCCTTTCGGACTCATCAGGGAAAGTACACACTTTCCGACGGTGCTCCCCATA

>ELVd L1R3
GGGTGGTGTGTGCCACCCCTGATGAGACCGAAAGGTCGAAATGGGGTTTCGCCATCAAACCCTTACTTTGTAAGT
GTGGTTCGGCGAATGTACCGTTTCGTCCTTTCGGACTCATCAGGGAAAGTACACACTTTCCGACGGTGGGTTCGT
CGATAGAGGACGACCTCTCCCCATA

>ELVd L2R2
GGGTGGTGTGTGCCACCCCTGATGAGACCGAAAGGTCGAAATGGGGTTTCGCCATGGGTCGGGACTTTAAATTCG
GAGGATTCGTCCTTTAAACGTTCCTCCAAGAGTCCCTTCCCCACGGCGAATGTACCGTTTCGTCCTTTCGGACTC
ATCAGGGAAAGTACACACTTTCCGACGGTGGGTTCGTCGACCTCTTTTGGATTGCTCCGGCCTTCCAGGAGAGAT
AGAGGACGACCTCTCCCCATA

>ELVd L3R1
GGGTGGTGTGTGCCACCCCTGATGAGACCGAAAGGTCGAAATGGGGTTTCGCCATTCGGCGAATGTACCGTTTCG
TCCTTTCGGACTCATCAGGGAAAGTACACACTTTCCGACGGTGGGTTCGTCGACACCTCTCCCCCTCCCAGGTAC
TATCCCCTTTCAAGGATGTGTTCCCTAGGAGGGTGGGTGTACATAGAGGACGACCTCTCCCCATA

>ELVd L3R3
GGGTGGTGTGTGCCACCCCTGATGAGACCGAAAGGTCGAAATGGGGTTTCGCCATTCGGCGAATGTACCGTTTCG
TCCTTTCGGACTCATCAGGGAAAGTACACACTTTCCGACGGTGGGTTCGTCGATAGAGGACGACCTCTCCCCATA

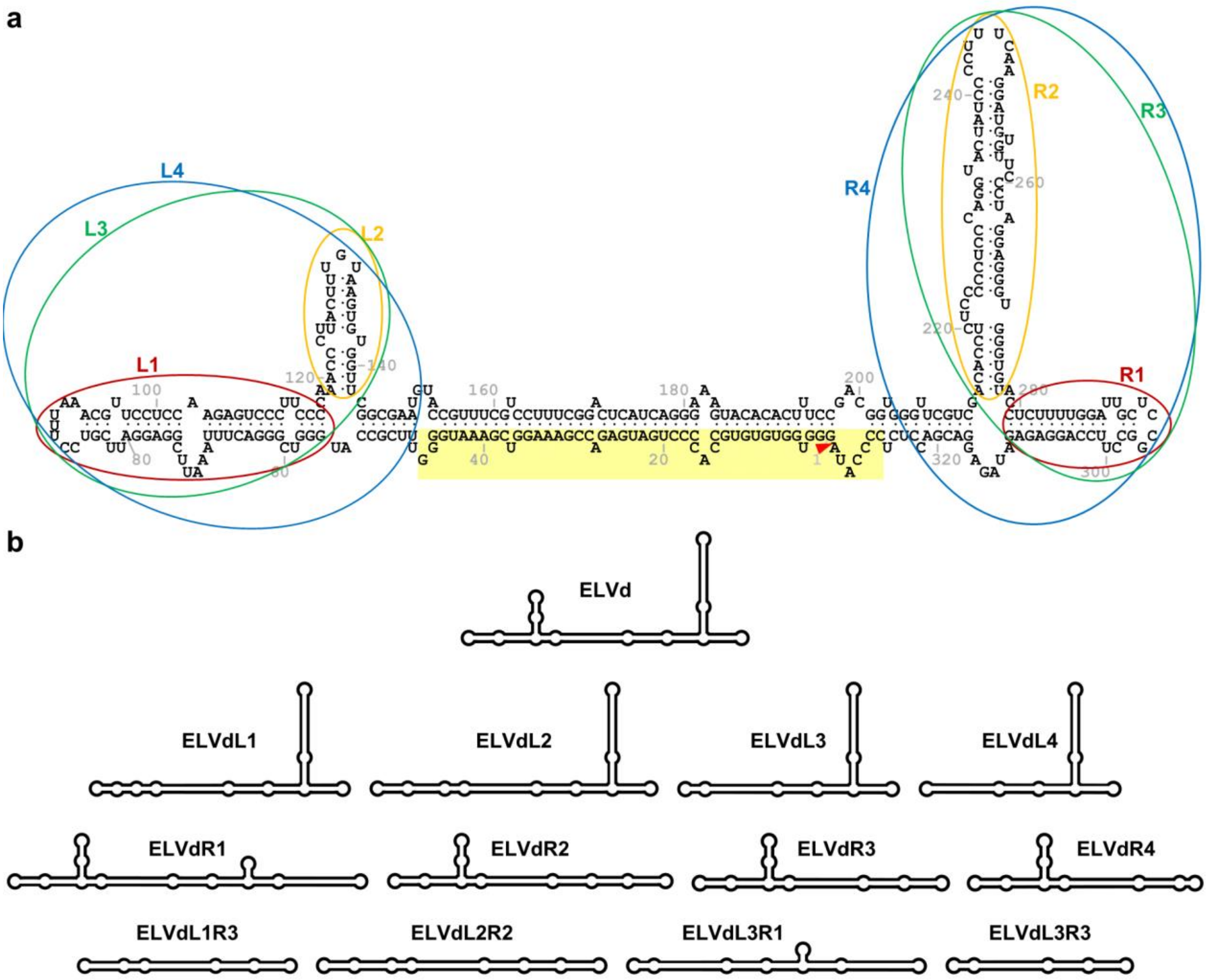

Figure S9. ELVd deleted forms assayed in the viroid-derived system to produce recombinant RNA in *E. coli*. Scheme of the deletions created on the ELVd RNA molecule and assayed in the *E. coli* system to overproduce recombinant RNA. (**a**) Structural domains of the folded ELVd molecule deleted at the left (L1, L2, L3 and L4) and right (R1, R2, R3 and R4) part of the molecule. (**b**) Schematic representation of full-length ELVd RNA and the resulting ELVd forms with single deletions at the left (ELVd L1, L2, L3 and L4), at the right (ELVd R1, R2, R3 and R4), and with double deletions at both sides (ELVd L1R3, L2R2, L3R1 and L3R3). RNA secondary structures of minimum free energy were calculated using the Mfold web server (http://unafold.rna.albany.edu/?q=mfold/RNA-Folding-Form).

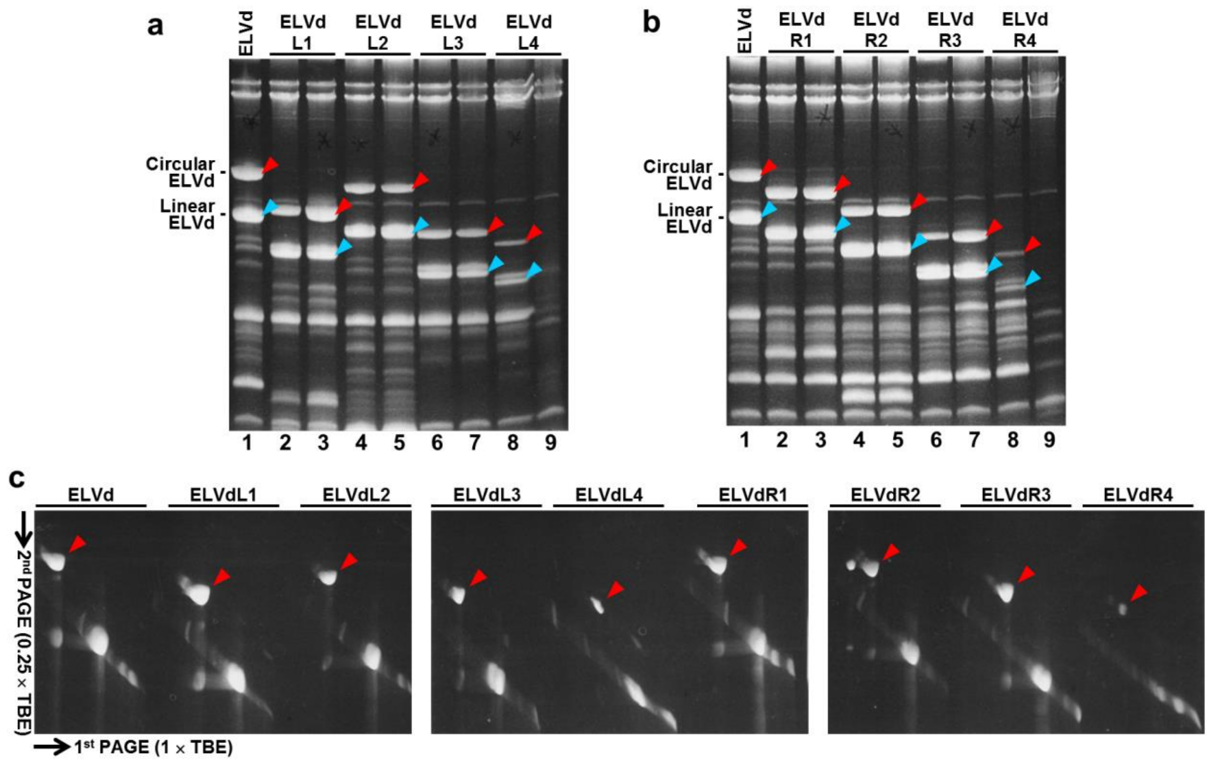

Figure S10. Analysis of the RNA that accumulated in *E. coli* that co-expressed eggplant tRNA ligase and deleted forms of ELVd RNA. Aliquots of the cultures were taken at 8 h post-induction of tRNA ligase expression. Purified RNA was separated by single (**a** and **b**) or double denaturing PAGE (**c**) and stained with ethidium bromide. (**a** and **b**) Lanes 1, RNAs from *E. coli* that expressed full-length ELVd. (**a**) Lanes 2 to 9, RNAs from two independent *E. coli* clones that expressed ELVd forms (see Supplementary Fig. S9) with deletions at the left side of the molecule, as indicated. (**b**) Lanes 2 to 9, RNAs from two independent *E. coli* clones that expressed ELVd forms with deletions at the right side of the molecule, as indicated. The positions of the circular and linear forms of full-length ELVd RNA are indicated on the left of both panels. The positions of the different circular and linear deleted ELVd RNAs are indicated by red and blue arrowheads, respectively. (**c**) RNAs from *E. coli* that expressed full-length ELVd RNA and ELVd deleted forms L1, L2, L3, L4, R1, R2, R3 and R4, as indicated. The directions of migration of the two electrophoretic separations are indicated. The positions of the corresponding circular RNAs are indicated by a red arrowhead. *E. coli* cultures were grown at 37ºC in LB medium and induced at DO_{600} 0.1 with 0.1 mM IPTG. Each lane contains an aliquot of RNA corresponding to 0.8 ml of culture.

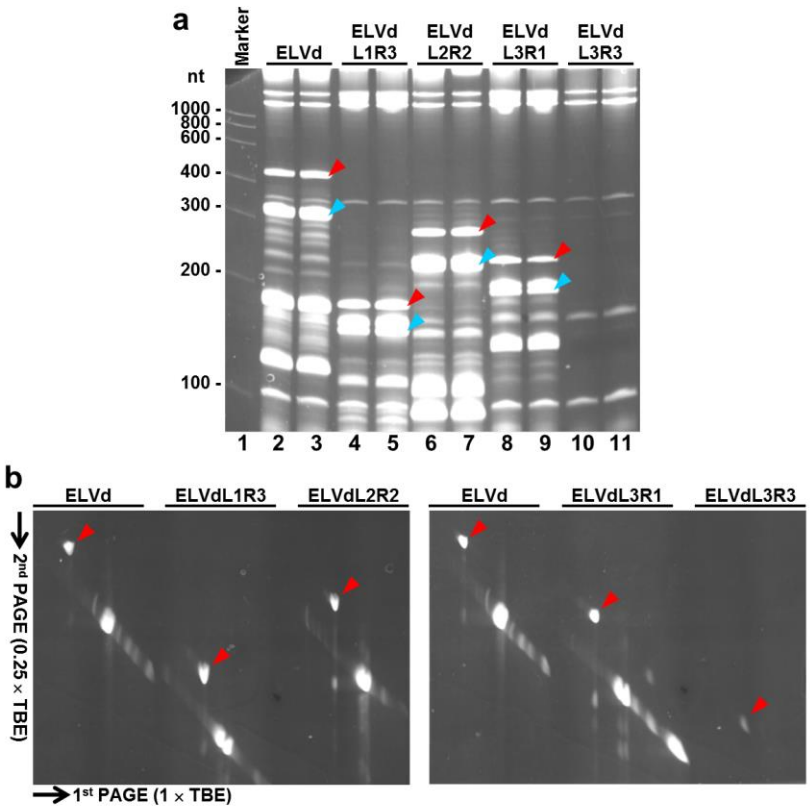

Figure S11. Analysis of the RNA that accumulated in *E. coli* that co-expressed eggplant tRNA ligase and double-deleted ELVd RNA forms. Aliquots of the cultures were taken at 8 h post-induction, RNA purified from the cells and separated by single dimension (**a**) or double dimension (**b**) denaturing PAGE. Gels were stained with ethidium bromide. (**a**) Lane 1, RNA marker with the sizes of the standards indicated on the left in nt; lanes 2 to 11, RNAs from two independent *E. coli* clones that expressed full-length ELVd (lanes 2 and 3) and the ELVd deleted forms L1R3 (lanes 4 and 5), L2R2 (lanes 6 and 7), L3R1 (lanes 8 and 9) and L2R2 (lanes 10 and 11). The positions of the corresponding circular and linear RNAs are indicated by red and blue arrowheads, respectively. (**b**) RNAs form *E. coli* clones that expressed full-length ELVd and ELVd forms with double deletion (L1R3, L2R2, L3R1 and L3R3), as indicated. The directions of migration of the two electrophoretic separations are indicated. The positions of the corresponding circular RNAs are indicated by a red arrowhead. *E. coli* cultures were grown at 37ºC in LB medium and induced at DO_{600} 0.1 with 0.1 mM IPTG. Each lane contains an aliquot of RNA corresponding to 0.8 ml of culture.

Figure S12. Sequence and elements of plasmid p15LtRnlSm used to constitutively express eggplant tRNA ligase in *E. coli*.

```
>p15LtRnlSm (5415 bp)
CGATGCTTCTTTGAGCGAACGATCAAAAATAAGTGCCTTCCCATCAAAAAAATATTCTCAACATAAAAAACTTTG
TGTAATACTTGTAACGCTGGGGAGACCACAACGGTTTCCCTCTAGAAATAATTTTGTTTAACTTTAAGAAGGAGA
TATACCATGTCGGTTTCGCATAGGGTCATTTACTCTTTCACTCATTACAAACTCTATAATCTCTCTTCTTCTTTA
TCATCTTTGCCTTCTAGAATCTTCTTCCCTTTTCAATCTCCTTCCTTTCACACGTTCTCTTCACTCATGCCCAAC
AATCAGGAAAGGGGTGGTTATGAAGGAAAAAAATGGCAAGTGAGGCCAAGTTCCAATAGGGTACCAGGCTCGTCT
TCAAATGTGGAACCTGTATCTGCTGCAACTGCTGAAGCCATTACCGACCGTCTAAAGTCCGTGGATATTACTGAA
AGTGGTGCACAGTCTAGTGTTCCAGTCACATCTCTTCAGTTTGGCAGCGTTGGATTAGCACCCCAGTCACCTGTG
CAACATCAAAAAGTAATCTGGAAACCCAAATCATATGGAACAGTGTCTGGAGCCCCAGTGGTTGAAGCTGGAAAA
ACACCAGTTGAACAAAAAAGTGCTCTTTTAAGTAAATTATTCAAGGGTAATTTATTGGAAAATTTTACTGTAGAT
AACTCAACATTCTCGAGAGCCCAAGTAAGGGCCACTTTCTACCCAAAATTTGAGAATGAGAAATCAGATCAGGAG
ATCAGGACAAGGATGATAGAGATGGTCTCCAAAGGCTTGGCTATAGTCGAGGTCACACTTAAGCATTCTGGATCT
CTTTTTATGTATGCTGGGCATGAAGGTGGAGCATATGCCAAGAATAGCTTCGGGAATATCTATACTGCCGTTGGC
GTCTTTGTTCTTGGACGGATGTTTCGTGAGGCATGGGGAACTAAAGCAAGCAAGAAGCAAGCAGAGTTCAATGAG
TTTCTTGAGCGCAATCGTATGTGCATATCAATGGAGTTGGTCACGGCAGTGTTGGGGGACCACGGACAACGCCCA
CGAGATGATTATGCGGTTGTGACTGCAGTCACGGAGTTGGGAAATGGAAAACCAACTTTCTATTCAACTCCCGAT
GTAATTGCTTTTTGCAGGGAATGGCGATTACCAACAAATCATGTATGGCTGTTCTCAACAAGGAAATCAGTGACT
TCCTTCTTTGCTGCGTATGATGCACTTTGCGAGGAAGGTACAGCAACCACCGTTTGCGAGGCTCTCAGCGAAGTT
GCTGATATTTCTGTACCTGGATCAAAAGACCATATAAAAGTGCAGGGTGAAATTTTGGAGGGTCTCGTGGCCCGC
ATCGTAAAACGTGAGAGCTCAGAGCATATGGAGCGGGTTCTGAGAGATTTTCCTCCTCCGCCATCAGAGGGTGAG
GGTTTGGACCTGGGACCTACGTTACGTGAAATTTGTGCTGCAAACAGATCAGAAAAGCAGCAAATAAAGGCACTT
CTTCAGAGTGCTGGCACGGCTTTCTGCCCGAATTATTTGGACTGGTTTGGAGATGAAAACTCTGGTTCACATTCA
AGAAATGCTGATCGATCTGTTGTCTCAAAGTTCTTACAATCACATCCTGCTGATCTTTATACAGGAAAAATACAG
GAAATGGTTCGCTTGATGAGGGAAAAGCGCTTTCCTGCTGCTTTCAAGTGTCATTATAACTTACATAAAATTAAT
GATGTATCGAGTAACAACCTGCCTTTCAAAATGGTGATCCATGTATATAGTGATTCAGGCTTCCGCCGGTACCAG
AAAGAGATGAGGCACAAACCAGGACTATGGCCTTTGTATCGAGGCTTTTTTGTTGACCTGGATTTATTCAAGGTC
AATGAGAAGAAAACTGCTGAAATGGCAGGAAGCAACAATCAAATGGTAAAAAATGTGGAAGAGGACAACAGTTTA
GCTGATGAAGATGCAAATCTGATGGTCAAGATGAAATTTCTTACTTACAAGTTGAGAACTTTTTTGATCCGTAAT
GGCTTGTCGACTCTTTTCAAAGAAGGACCTTCTGCGTATAAGTCTTATTACCTGAGGCAAATGAAAATTTGGAAT
ACTTCAGCAGCCAAGCAACGAGAACTCAGCAAGATGCTTGATGAATGGGCAGTATATATACGCAGAAAATATGGG
AACAAACCATTGTCATCATCCACATACCTAAGTGAAGCTGAGCCTTTCCTTGAACAATATGCAAAGCGTAGTCCA
CAAAATCATGCTTTGATAGGATCTGCTGGAAATTTTGTCAAAGTTGAAGATTTCATGGCTATTGTTGAAGGAGAA
GATGAAGAGGGTGATCTCGAGCCTGCGAAAGATATTGCTCCTTCAAGCCCTAGTATTTCCACCAGAGACATGGTG
GCAAAGAATGAGGGTCTCATTATTTTCTTTCCAGGAATACCAGGTTGTGCTAAATCTGCACTTTGTAAGGAAATA
CTGAATGCTCCAGGAGGGCTTGGAGATGATCGACCAGTTAACAGTTTAATGGGTGATCTTATTAAAGGTAGATAT
TGGCAAAAAGTTGCTGATGAACGTCGAAGAAAACCTTACTCGATCATGCTTGCTGACAAGAATGCACCAAATGAG
GAAGTATGGAAACAAATTGAGAACATGTGCCTAAGCACCGGAGCATCTGCTATTCCAGTTATACCTGATTCAGAA
GGAACTGAAACTAATCCATTCTCTATTGATGCACTTGCGGTTTTTATATTCCGAGTACTTCACCGTGTCAATCAT
CCGGGAAATCTTGACAAGTCATCTCCAAATGCTGGATATGTGATGCTTATGTTTTATCACCTTTATGATGGAAAG
AGCCGTCAGGAGTTCGAGAGTGAGCTTATTGAACGTTTTGGATCGCTTGTCAGAATTCCTGTACTGAAACCTGAG
AGGTCTCCTCTTCCGGATTCTGTGAGGTCTATTATCGAGGAGGGACTCAGTCTGTACAGACTTCATACAACGAAA
CATGGAAGATTGGAGTCTACAAAAGGGACATATGTACAAGAGTGGGTTAAATGGGAGAAGCAATTGAGAGATATT
CTACTTGGAAATGCAGACTATCTCAATTCAATACAGGTTCCATTTGAATTTGCCGTTAAAGAAGTCCTTGAACAA
CTGAAAGTTATTGCGAGGGGCGAATATGCAGTGCCTGCTGAGAAGAGGAAGCTAGGATCCATTGTATTCGCCGCT
ATCAGCCTGCCAGTTCCAGAAATTCTAGGTCTTCTAAATGATCTAGCAAAGAAAGATCCAAAGGTTGGCGATTTC
ATTAAGGACAAGAGCATGGAGAGCAGCATTCAGAAGGCCCATCTTACCCTGGCTCACAAGAGAAGTCACGGTGTC
ACTGCAGTTGCCAATTACGGTTCCTTTCTTCATCAAAAGGTGCCAGTAGACGTGGCTGCTTTGTTGTTCTCCGAT
AAATTGGCTGCACTAGAAGCTGAGCCTGGCTCTGTTGAAGGTGAAAAGATCAATTCTAAAAACTCATGGCCCCAT
ATCACATTATGGTCTGGTGCAGGAGTTGCCGCAAAAGATGCCAATACACTACCACAGTTACTTTCCCAAGGGAAG
GCTACCCGCATTGATATAAATCCACCGGTCACTATAACTGGCACTCTCGAATTCTTTCACCACCACCACCACCAC
TGAGATCCGGCTGCTAACAAAGCCCGAAAGGAAGCTGAGTTGGCTGCTGCCACCGCTGAGCAATAACTAGCATAA
CCCCTTGGGGCCTCTAAACGGGTCTTGAGGGGTTTTTTGCTGAAAGGAGATCTGGCGGGCCCGCGCTAGCGGAG
TGTATACTGGCTTACTATGTTGGCACTGATGAGGGTGTCAGTGAAGTGCTTCATGTGGCAGGAGAAAAAAGGCTG
CACCGGTGCGTCAGCAGAATATGTGATACAGGATATATTCCGCTTCCTCGCTCACTGACTCGCTACGCTCGGTCG
TTCGACTGCGGCGAGCGGAAATGGCTTACGAACGGGGCGGAGATTTCCTGGAAGATGCCAGGAAGATACTTAACA
GGGAAGTGAGAGGGCCGCGGCAAAGCCGTTTTTCCATAGGCTCCGCCCCCCTGACAAGCATCACGAAATCTGACG
CTCAAATCAGTGGTGGCGAAACCCGACAGGACTATAAAGATACCAGGCGTTTCCCCCTGGCGGCTCCCTCGTGCG
```

CTCTCCTGTTCCTGCCTTTCGGTTTACCGGTGTCATTCCGCTGTTATGGCCGCGTTTGTCTCATTCCACGCCTGA
CACTCAGTTCCGGGTAGGCAGTTCGCTCCAAGCTGGACTGTATGCACGAACCCCCCGTTCAGTCCGACCGCTGCG
CCTTATCCGGTAACTATCGTCTTGAGTCCAACCCGGAAAGACATGCAAAAGCACCACTGGCAGCAGCCACTGGTA
ATTGATTTAGAGGAGTTAGTCTTGAAGTCATGCGCCGGTTAAGGCTAAACTGAAAGGACAAGTTTTGGTGACTGC
GCTCCTCCAAGCCAGTTACCTCGGTTCAAAGAGTTGGTAGCTCAGAGAACCTTCGAAAAACCGCCCTGCAAGGCG
GTTTTTTCGTTTTCAGAGCAAGAGATTACGCGCAGACCAAAACGATCTCAAGAAGATCATCTTATTAATTACGCC
CCGCCCTGCCACTCATCGCAGTACTGTTGTAATTCATTAAGCATTCTGCCGACATGGAAGCCATCACAGACGGCA
TGATGAACCTGAATCGCCAGCGGCATCAGCACCTTGTCGCCTTGCGTATAATATTTGCCCATGGTGAAAACGGGG
GCGAAGAAGTTGTCCATATTGGCCACGTTTAAATCAAAACTGGTGAAACTCACCCAGGGATTGGCTGAGACGAAA
AACATATTCTCAATAAACCCTTTAGGGAAATAGGCCAGGTTTTCACCGTAACACGCCACATCTTGCGAATATATG
TGTAGAAACTGCCGGAAATCGTCGTGGTATTCACTCCAGAGCGATGAAAACGTTTCAGTTTGCTCATGGAAAACG
GTGTAACAAGGGTGAACACTATCCCATATCACCAGCTCACCGTCTTTCATTGCCATACGGAATTCCGGATGAGCA
TTCATCAGGCGGGCAAGAATGTGAATAAAGGCCGGATAAAACTTGTGCTTATTTTTCTTTACGGTCTTTAAAAAG
GCCGTAATATCCAGCTGAACGGTCTGGTTATAGGTACATTGAGCAACTGACTGAAATGCCTCAAAATGTTCTTTA
CGATGCCATTGGGATATATCAACGGTGGTATATCCAGTGATTTTTTTCTCCAT*TTTAGCTTCCTTAGCTCCTGAA*
*AATCTCGATAACTCAAAAAATACGCCCGGTAGTGATCTTATTTCATTATGGTGAAAGTTGGAA*CCCGGGGGCGGC
GGCCGCCCGCGAAAT

E. coli **murein lipoprotein promoter** is in red. Eggplant tRNA ligase (JX0225157) in black with the theoretical amino-terminal **transit peptide** on green background and the carboxy-terminal **hexahistidine tag** in blue. **Stop codon** in red. **T7 bacteriophage terminator** on red background. **p15A replication origin** in gray and **chloramphenicol resistance gene** (reverse orientation) in blue (***promoter*** in italics).

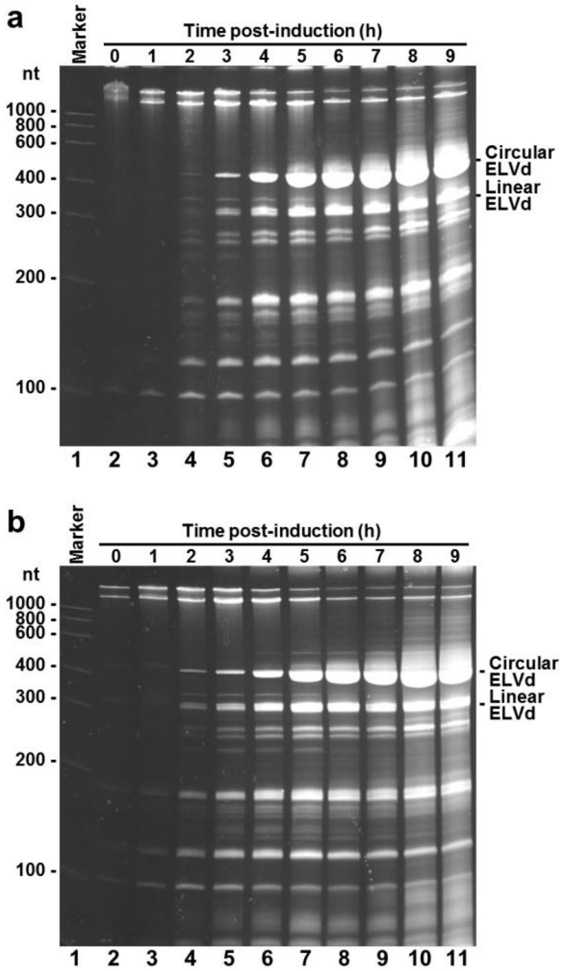

Figure S13. Time-course analysis of the RNA that accumulated in *E. coli* that co-expressed ELVd RNA and eggplant tRNA ligase under the control of (a) IPTG-inducible T7 bacteriophage RNA polymerase and (b) constitutive *E. coli* murein lipoprotein promoters. Aliquots from the cultures were taken at different time points, and RNAs purified and separated by denaturing PAGE. Gels were stained with ethidium bromide. Lanes 1, RNA marker with the sizes of the standards indicated on the left in nt; lanes 2 to 11, RNAs from aliquots taken at 0, 1, 2, 3, 4, 5, 6, 7, 8 and 9 h post-induction. The positions of the circular and linear ELVd RNAs are indicated on the right. *E. coli* cultures were grown at 37ºC in TB medium and induced with 0.1 mM ITPG (**a**) or not induced (**b**) at OD_{600} 0.1. Each lane contains an aliquot of RNA corresponding to 0.2 ml of culture.

Figure S14. Sequence and elements of plasmids pLELVd-Spinach and pLELVdL1R3-Spinach.

>pLELVd-Spinach (2148 bp)
CGATGCTTCTTTGAGCGAACGATCAAAAATAAGTGCCTTCCCATCAAAAAAATATTCTCAACATAAAAAACTTTG
TGTAATACTTGTAACGCTGCCCCATAGGGTGGTGTGTGCCACCCCTGATGAGACCGAAAGGTCGAAATGGGGTTT
CGCCATGGGTCGGGACTTTAAATTCGGAGGATTCGTCCTTTAAACGTTCCTCCAAGAGTCCCTTCCCCAAACCCT
TACTTTGTAAGTGTGGTTCGGCGAATGTACCGTTTCGTCCTTTCGGACTCATCAGGGAAAGTACACACTTTCCGA
CGGTGGGTTCGTCGACACCTCTCCCCCTCCCAGGTACTATCCCCTTGACGCAACTGAATGAAATGGTGAAGGACG
GGTCCAGGTGTGGCTGCTTCGGCAGTGCAGCTTGTTGAGTAGAGTGTGAGCTCCGTAACTAGTCGCGTCTCAAGG
ATGTGTTCCCTAGGAGGGTGGGTGTACCTCTTTTGGATTGCTCCGGCCTTCCAGGAGAGATAGAGGACGACCTCT
CCCCATAGGGTGGTGTGTGCCACCCCTGATGAGACCGAAAGGTCGAAATGGGGGAAATCATCCTTAGCGAAAGCT
AAGGATTTTTTTTATCTGAAATGCGTTGCTGGCGTTTTTCCATAGGCTCCGCCCCCCTGACGAGCATCACAAAAA
TCGACGCTCAAGTCAGAGGTGGCGAAACCCGACAGGACTATAAAGATACCAGGCGTTTCCCCCTGGAAGCTCCCT
CGTGCGCTCTCCTGTTCCGACCCTGCCGCTTACCGGATACCTGTCCGCCTTTCTCCCTTCGGGAAGCGTGGCGCT
TTCTCATAGCTCACGCTGTAGGTATCTCAGTTCGGTGTAGGTCGTTCGCTCCAAGCTGGGCTGTGTGCACGAACC
CCCCGTTCAGCCCGACCGCTGCGCCTTATCCGGTAACTATCGTCTTGAGTCCAACCCGGTAAGACACGACTTATC
GCCACTGGCAGCAGCCACTGGTAACAGGATTAGCAGAGCGAGGTATGTAGGCGGTGCTACAGAGTTCTTGAAGTG
GTGGCCTAACTACGGCTACACTAGAAGGACAGTATTTGGTATCTGCGCTCTGCTGAAGCCAGTTACCTTCGGAAA
AAGAGTTGGTAGCTCTTGATCCGGCAAACAAACCACCGCTGGTAGCGGTGGTTTTTTTGTTTGCAAGCAGCAGAT
TACGCGCAGAAAAAAAGGATCTCAAGAAGATCCTTTTTACCAATGCTTAATCAGTGAGGCACCTATCTCAGCGAT
CTGTCTATTTCGTTCATCCATAGTTGCCTGACTCCCCGTCGTGTAGATAACTACGATACGGGAGGGCTTACCATC
TGGCCCCAGTGCTGCAATGATACCGCGAGAGCCACGCTCACCGGCTCCAGATTTATCAGCAATAAACCAGCCAGC
CGGAAGGGCCGAGCGCAGAAGTGGTCCTGCAACTTTATCCGCCTCCATCCAGTCTATTAATTGTTGCCGGGAAGC
TAGAGTAAGTAGTTCGCCAGTTAATAGTTTGCGCAACGTTGTTGCCATTGCTACAGGCATCGTGGTGTCACGCTC
GTCGTTTGGTATGGCTTCATTCAGCTCCGGTTCCCAACGATCAAGGCGAGTTACATGATCCCCCATGTTGTGCAA
AAAAGCGGTTAGCTCCTTCGGTCCTCCGATCGTTGTCAGAAGTAAGTTGGCCGCAGTGTTATCACTCATGGTTAT
GGCAGCACTGCATAATTCTCTTACTGTCATGCCATCCGTAAGATGCTTTTCTGTGACTGGTGAGTACTCAACCAA
GTCATTCTGAGAATAGTGTATGCGGCGACCGAGTTGCTCTTGCCCGGCGTCAATACGGGATAATACCGCGCCACA
TAGCAGAACTTTAAAAGTGCTCATCATTGGAAAACGTTCTTCGGGGCGAAAACTCTCAAGGATCTTACCGCTGTT
GAGATCCAGTTCGATGTAACCCACTCGTGCACCCAACTGATCTTCAGCATCTTTTACTTTCACCAGCGTTTCTGG
GTGAGCAAAAACAGGAAGGCAAAATGCCGCAAAAAAGGGAATAAGGGCGACACGGAAATGTTGAATACTCATACT
CTTCCTTTTTCAATATTATTGAAGCATTTATCAGGGTTATTGTCTCAT

E. coli **murein lipoprotein promoter** is in red and **ELVd** cDNA **(**C327 to G46 of AJ536613) is in black with the repeated hammerhead ribozyme domain on yellow background. cDNA coding for the RNA aptamer **Spinach** in green inserted between T245 and T246 of ELVd cDNA. Ribozymes self-cleavage sites are underlined. *E. coli* ribosomal **rrnC terminator** is in fuchsia. **pUC replication origin** is in gray and **ampicillin resistance gene** (inverse orientation) on gray background (**promoter** on dark gray background).

>pLELVdL3R1-Spinach (2030 bp)
CGATGCTTCTTTGAGCGAACGATCAAAAATAAGTGCCTTCCCATCAAAAAAATATTCTCAACATAAAAAACTTTG
TGTAATACTTGTAACGCTGCCCCATAGGGTGGTGTGTGCCACCCCTGATGAGACCGAAAGGTCGAAATGGGGTTT
CGCCATTCGGCGAATGTACCGTTTCGTCCTTTCGGACTCATCAGGGAAAGTACACACTTTCCGACGGTGGGTTCG
TCGACACCTCTCCCCCTCCCAGGTACTATCCCCTTGACGCAACTGAATGAAATGGTGAAGGACGGGTCCAGGTGT
GGCTGCTTCGGCAGTGCAGCTTGTTGAGTAGAGTGTGAGCTCCGTAACTAGTCGCGTCTCAAGGATGTGTTCCCT
AGGAGGGTGGGTGTACATAGAGGACGACCTCTCCCCATAGGGTGGTGTGTGCCACCCCTGATGAGACCGAAAGGT
CGAAATGGGGGAAATCATCCTTAGCGAAAGCTAAGGATTTTTTTTATCTGAAATGCGTTGCTGGCGTTTTTCCAT
AGGCTCCGCCCCCCTGACGAGCATCACAAAAATCGACGCTCAAGTCAGAGGTGGCGAAACCCGACAGGACTATAA
AGATACCAGGCGTTTCCCCCTGGAAGCTCCCTCGTGCGCTCTCCTGTTCCGACCCTGCCGCTTACCGGATACCTG
TCCGCCTTTCTCCCTTCGGGAAGCGTGGCGCTTTCTCATAGCTCACGCTGTAGGTATCTCAGTTCGGTGTAGGTC
GTTCGCTCCAAGCTGGGCTGTGTGCACGAACCCCCCGTTCAGCCCGACCGCTGCGCCTTATCCGGTAACTATCGT
CTTGAGTCCAACCCGGTAAGACACGACTTATCGCCACTGGCAGCAGCCACTGGTAACAGGATTAGCAGAGCGAGG
TATGTAGGCGGTGCTACAGAGTTCTTGAAGTGGTGGCCTAACTACGGCTACACTAGAAGGACAGTATTTGGTATC
TGCGCTCTGCTGAAGCCAGTTACCTTCGGAAAAAGAGTTGGTAGCTCTTGATCCGGCAAACAAACCACCGCTGGT
AGCGGTGGTTTTTTTGTTTGCAAGCAGCAGATTACGCGCAGAAAAAAAGGATCTCAAGAAGATCCTTTTTACCAA
TGCTTAATCAGTGAGGCACCTATCTCAGCGATCTGTCTATTTCGTTCATCCATAGTTGCCTGACTCCCCGTCGTG
TAGATAACTACGATACGGGAGGGCTTACCATCTGGCCCCAGTGCTGCAATGATACCGCGAGAGCCACGCTCACCG

GCTCCAGATTTATCAGCAATAAACCAGCCAGCCGGAAGGGCCGAGCGCAGAAGTGGTCCTGCAACTTTATCCGCC
TCCATCCAGTCTATTAATTGTTGCCGGGAAGCTAGAGTAAGTAGTTCGCCAGTTAATAGTTTGCGCAACGTTGTT
GCCATTGCTACAGGCATCGTGGTGTCACGCTCGTCGTTTGGTATGGCTTCATTCAGCTCCGGTTCCCAACGATCA
AGGCGAGTTACATGATCCCCCATGTTGTGCAAAAAAGCGGTTAGCTCCTTCGGTCCTCCGATCGTTGTCAGAAGT
AAGTTGGCCGCAGTGTTATCACTCATGGTTATGGCAGCACTGCATAATTCTCTTACTGTCATGCCATCCGTAAGA
TGCTTTTCTGTGACTGGTGAGTACTCAACCAAGTCATTCTGAGAATAGTGTATGCGGCGACCGAGTTGCTCTTGC
CCGGCGTCAATACGGGATAATACCGCGCCACATAGCAGAACTTTAAAAGTGCTCATCATTGGAAAACGTTCTTCG
GGGCGAAAACTCTCAAGGATCTTACCGCTGTTGAGATCCAGTTCGATGTAACCCACTCGTGCACCCAACTGATCT
TCAGCATCTTTTACTTTCACCAGCGTTTCTGGGTGAGCAAAAACAGGAAGGCAAAATGCCGCAAAAAAGGGAATA
AGGGCGACACGGAAATGTTGAATACTCATACTCTTCCTTTTTCAATATTATTGAAGCATTTATCAGGGTTATTGT
CTCAT

E. coli **murein lipoprotein promoter** is in red and **ELVd L3R1** cDNA (see Supplementary Dataset 8) is in black with the repeated hammerhead ribozyme domain on yellow background. cDNA coding for the RNA aptamer **Spinach** in green inserted between T245 and T246 of ELVd-AJ536613 cDNA. Ribozymes self-cleavage sites are underlined. *E. coli* ribosomal **rrnC terminator** is in fuchsia. **pUC replication origin** is in gray and **ampicillin resistance gene** (inverse orientation) on gray background (**promoter** on dark gray background).

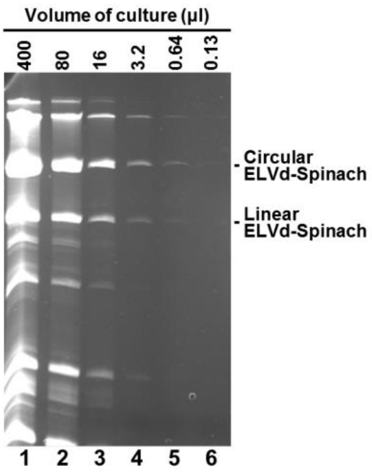

Figure S15. Quantification of the chimeric ELVd-Spinach RNA produced with the viroid-derived system in *E. coli*. The RNA extracted from an aliquot of the culture taken at 12 h post-induction (see Fig. 3a) was subjected to serial dilutions. Samples were separated by denaturing PAGE. The gel was stained with ethidium bromide and the intensities of the bands compared with those in a gel photographed in the same conditions containing known amounts of an RNA standard (*E. coli* 5S rRNA, Supplementary Fig. S7b). Lanes 1 to 6, serial dilutions of the RNA corresponding to 400, 80, 16, 3.2, 0.64 and 0.13 µl of the original culture, respectively. The positions of the circular and linear ELVd-Spinach RNAs are indicated on the right. *E. coli* cultures were grown at 37ºC in TB medium and induced with 0.1 mM ITPG at DO_{600} 0.1.

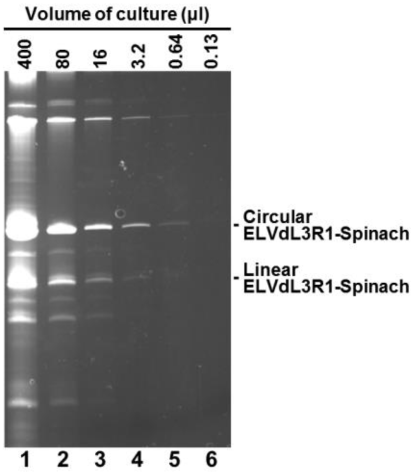

Figure S16. Quantification of the chimeric ELVd L3R1-Spinach RNA produced in *E. coli* using the viroid-derived system. The RNA extracted from the aliquot of the *E. coli* culture taken at 12 h post-induction was subjected to 1/5 serial dilutions and separated by denaturing PAGE. The gel was stained with ethidium bromide and the intensities of the bands compared with those in a gel photographed in the same conditions containing known amounts of an RNA standard (*E. coli* 5S rRNA, Supplementary Fig. S7b). Lanes 1 to 6, RNAs corresponding to 400, 80, 16, 3.2, 0.64 and 0.13 µl of the original culture, respectively. The positions of the circular and linear ELVdL3R1-Spinach RNAs are indicated on the right.

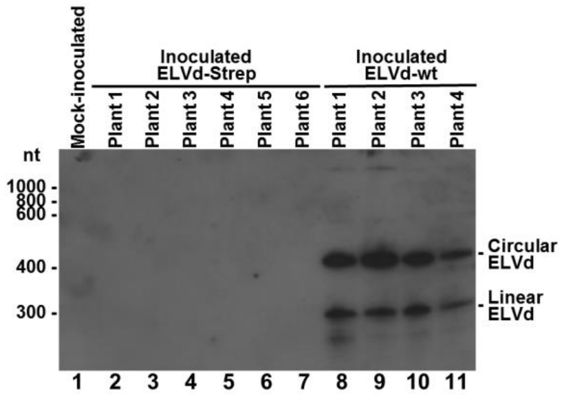

Figure S17. Infectivity of chimeric ELVd-Spinach RNA. Eggplants were mock-inoculated or inoculated with chimeric ELVd-Spinach or wild-type ELVd RNA by means of *Agrobacterium tumefaciens*. Total RNAs were purified from systemic tissue of inoculated plants 45 days post-inoculation, separated by denaturing PAGE, transferred to a membrane and hybridized with an ELVd-specific radioactive RNA probe. The membrane was finally autoradiographed. Lane 1, RNA from a mock-inoculated eggplant; lanes 2 to 7, RNAs from 6 independent eggplants agroinoculated with ELVd-Spinach; lanes 8 to 11, RNAs from 4 independent eggplants agroinoculated with wild-type ELVd. The positions and sizes (in nt) of RNA markers are indicated on the left and the positions of monomeric circular and linear ELVd RNAs on the right of the panel.

Figure S18. Sequences and elements of plasmids pLELVd-hairpin 100, 80, 60 and 40.

>pLELVd-hairpin 100 (2150 bp)
CGATGCTTCTTTGAGCGAACGATCAAAAATAAGTGCCTTCCCATCAAAAAAATATTCTCAACATAAAAAACTTTG
TGTAATACTTGTAACGCTGCCCCATAGGGTGGTGTGTGCCACCCCTGATGAGACCGAAAGGTCGAAATGGGGTTT
CGCCATGGGTCGGACTTTAAATTCGGAGGATTCGTCCTTTAAACGTTCCTCCAAGAGTCCCTTCCCCAAACCCT
TACTTTGTAAGTGTGGTTCGGCGAATGTACCGTTTCGTCCTTTCGGACTCATCAGGGAAAGTACACACTTTCCGA
CGGTGGGTTCGTCGACACCTCTCCCCCTCCCAGGTACTATCCCCTTGCGAATTTGTTTTCGTATTTGGATACCTT
CTTCAAGGTTGGTTATGGGTATACTTCATACCAACCTTGAAGAAGGTATCCAAATACGAAAACAAATTCGCTCAA
GGATGTGTTCCCTAGGAGGGTGGGTGTACCTCTTTTGGATTGCTCCGGCCTTCCAGGAGAGATAGAGGACGACCT
CTCCCCATAGGGTGGTGTGTGCCACCCCTGATGAGACCGAAAGGTCGAAATGGGGGAAATCATCCTTAGCGAAAG
CTAAGGATTTTTTTTATCTGAAATGCGTTGCTGGCGTTTTTCCATAGGCTCCGCCCCCCTGACGAGCATCACAAA
AATCGACGCTCAAGTCAGAGGTGGCGAAACCCGACAGGACTATAAAGATACCAGGCGTTTCCCCCTGGAAGCTCC
CTCGTGCGCTCTCCTGTTCCGACCCTGCCGCTTACCGGATACCTGTCCGCCTTTCTCCCTTCGGGAAGCGTGGCG
CTTTCTCATAGCTCACGCTGTAGGTATCTCAGTTCGGTGTAGGTCGTTCGCTCCAAGCTGGGCTGTGTGCACGAA
CCCCCCGTTCAGCCCGACCGCTGCGCCTTATCCGGTAACTATCGTCTTGAGTCCAACCCGGTAAGACACGACTTA
TCGCCACTGGCAGCAGCCACTGGTAACAGGATTAGCAGAGCGAGGTATGTAGGCGGTGCTACAGAGTTCTTGAAG
TGGTGGCCTAACTACGGCTACACTAGAAGGACAGTATTTGGTATCTGCGCTCTGCTGAAGCCAGTTACCTTCGGA
AAAAGAGTTGGTAGCTCTTGATCCGGCAAACAAACCACCGCTGGTAGCGGTGGTTTTTTTGTTTGCAAGCAGCAG
ATTACGCGCAGAAAAAAAGGATCTCAAGAAGATCCTTTTTACCAATGCTTAATCAGTGAGGCACCTATCTCAGCG
ATCTGTCTATTTCGTTCATCCATAGTTGCCTGACTCCCCGTCGTGTAGATAACTACGATACGGGAGGGCTTACCA
TCTGGCCCCAGTGCTGCAATGATACCGCGAGAGCCACGCTCACCGGCTCCAGATTTATCAGCAATAAACCAGCCA
GCCGGAAGGGCCGAGCGCAGAAGTGGTCCTGCAACTTTATCCGCCTCCATCCAGTCTATTAATTGTTGCCGGGAA
GCTAGAGTAAGTAGTTCGCCAGTTAATAGTTTGCGCAACGTTGTTGCCATTGCTACAGGCATCGTGGTGTCACGC
TCGTCGTTTGGTATGGCTTCATTCAGCTCCGGTTCCCAACGATCAAGGCGAGTTACATGATCCCCCATGTTGTGC
AAAAAAGCGGTTAGCTCCTTCGGTCCTCCGATCGTTGTCAGAAGTAAGTTGGCCGCAGTGTTATCACTCATGGTT
ATGGCAGCACTGCATAATTCTCTTACTGTCATGCCATCCGTAAGATGCTTTTCTGTGACTGGTGAGTACTCAACC
AAGTCATTCTGAGAATAGTGTATGCGGCGACCGAGTTGCTCTTGCCCGGCGTCAATACGGGATAATACCGCGCCA
CATAGCAGAACTTTAAAAGTGCTCATCATTGGAAAACGTTCTTCGGGGCGAAAACTCTCAAGGATCTTACCGCTG
TTGAGATCCAGTTCGATGTAACCCACTCGTGCACCCAACTGATCTTCAGCATCTTTTACTTTCACCAGCGTTTCT
GGGTGAGCAAAAACAGGAAGGCAAAATGCCGCAAAAAAGGGAATAAGGGCGACACGGAAATGTTGAATACTCATA
CTCTTCCTTTTTCAATATTATTGAAGCATTTATCAGGGTTATTGTCTCAT

E. coli **murein lipoprotein promoter** is in red and **ELVd** cDNA **(**C327 to G46 of AJ536613) is in black with the repeated hammerhead ribozyme domain on yellow background. cDNA coding for the **100-nt-long hairpin** structure in blue inserted between T245 and T246 of ELVd cDNA. The sequence corresponding to the **16-nt loop** is underlined. Ribozymes self-cleavage sites are underlined. *E. coli* ribosomal **rrnC terminator** is in fuchsia. **pUC replication origin** is in gray and **ampicillin resistance gene** (inverse orientation) on gray background (**promoter** on dark gray background).

In pLELVd-hairpin 80, 60 and 40, the sequences of the cDNAs coding for the different hairpins of 80, 60 and 40 nt are:

>pLELVd-hairpin 80
TTTCGTATTTGGATACCTTCTTCAAGGTTGGTTATGGGTATACTTCATACCAACCTTGAAGAAGGTATCCAAATA
CGAAA

>pLELVd-hairpin 60
GGATACCTTCTTCAAGGTTGGTTATGGGTATACTTCATACCAACCTTGAAGAAGGTATCC

>pLELVd-hairpin 40
TTCAAGGTTGGTTATGGGTATACTTCATACCAACCTTGAA

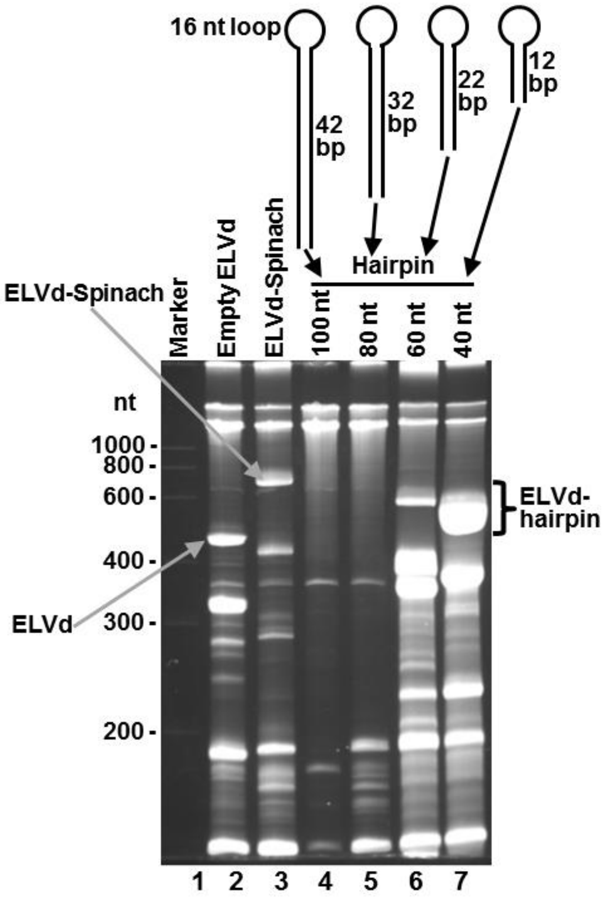

Supplementary Fig. S19. Production of recombinant RNAs that included extended hairpin structures using the viroid-derived system in *E. coli* BL21(DE3). RNAs were extracted from aliquots of *E. coli cultures* taken at 15:30 h post-inoculation. RNAs were separated by denaturing PAGE and stained with ethidium bromide. Lane 1, RNA marker with the sizes of the standards indicated on the left in nt; lanes 2 to 7, RNAs from *E. coli* transformed to express empty ELVd (lane 2), ELVd-Spinach (lane 3) and different ELVd forms that included hairpin RNAs of 100, 80, 60 and 40 nt as indicated (lanes 4 to 7). All *E. coli* were co-transformed with plasmid p15LtRnlSm. The positions of the circular empty ELVd and ELVd-Spinach are indicated on the left. The positions of some ELVd-hairpin RNAs are indicated on the right. *E. coli* cultures were grown at 37ºC in TB medium. Each lane contains an aliquot of RNA corresponding to 0.2 ml of culture.

Figure S20. Sequence and element of plasmid pLELVd-crRNA.

```
>pLELVd-crRNA (2115 bp)
CGATGCTTCTTTGAGCGAACGATCAAAAATAAGTGCCTTCCCATCAAAAAAATATTCTCAACATAAAAAACTTTG
TGTAATACTTGTAACGCTGCCCCATAGGGTGGTGTGTGCCACCCCTGATGAGACCGAAAGGTCGAAATGGGGTTT
CGCCATGGGTCGGGACTTTAAATTCGGAGGATTCGTCCTTTAAACGTTCCTCCAAGAGTCCCTTCCCCAAACCCT
TACTTTGTAAGTGTGGTTCGGCGAATGTACCGTTTCGTCCTTTCGGACTCATCAGGGAAAGTACACACTTTCCGA
CGGTGGGTTCGTCGACACCTCTCCCCCTCCCAGGTACTATCCCCTTTAATTTCTACTAAGTGTAGATCTGATGGT
CCATGTCTGTTACTCTAATTTCTACTAAGTGTAGATTCAAGGATGTGTTCCCTAGGAGGGTGGGTGTACCTCTTT
TGGATTGCTCCGGCCTTCCAGGAGAGATAGAGGACGACCTCTCCCCATAGGGTGGTGTGTGCCACCCCTGATGAG
ACCGAAAGGTCGAAATGGGGGAAATCATCCTTAGCGAAAGCTAAGGATTTTTTTTATCTGAAATGCGTTGCTGGC
GTTTTTCCATAGGCTCCGCCCCCCTGACGAGCATCACAAAAATCGACGCTCAAGTCAGAGGTGGCGAAACCCGAC
AGGACTATAAAGATACCAGGCGTTTCCCCCTGGAAGCTCCCTCGTGCGCTCTCCTGTTCCGACCCTGCCGCTTAC
CGGATACCTGTCCGCCTTTCTCCCTTCGGGAAGCGTGGCGCTTTCTCATAGCTCACGCTGTAGGTATCTCAGTTC
GGTGTAGGTCGTTCGCTCCAAGCTGGGCTGTGTGCACGAACCCCCCGTTCAGCCCGACCGCTGCGCCTTATCCGG
TAACTATCGTCTTGAGTCCAACCCGGTAAGACACGACTTATCGCCACTGGCAGCAGCCACTGGTAACAGGATTAG
CAGAGCGAGGTATGTAGGCGGTGCTACAGAGTTCTTGAAGTGGTGGCCTAACTACGGCTACACTAGAAGGACAGT
ATTTGGTATCTGCGCTCTGCTGAAGCCAGTTACCTTCGGAAAAAGAGTTGGTAGCTCTTGATCCGGCAAACAAAC
CACCGCTGGTAGCGGTGGTTTTTTTGTTTGCAAGCAGCAGATTACGCGCAGAAAAAAAGGATCTCAAGAAGATCC
TTTTTACCAATGCTTAATCAGTGAGGCACCTATCTCAGCGATCTGTCTATTTCGTTCATCCATAGTTGCCTGACT
CCCCGTCGTGTAGATAACTACGATACGGGAGGGCTTACCATCTGGCCCCAGTGCTGCAATGATACCGCGAGAGCC
ACGCTCACCGGCTCCAGATTTATCAGCAATAAACCAGCCAGCCGGAAGGGCCGAGCGCAGAAGTGGTCCTGCAAC
TTTATCCGCCTCCATCCAGTCTATTAATTGTTGCCGGGAAGCTAGAGTAAGTAGTTCGCCAGTTAATAGTTTGCG
CAACGTTGTTGCCATTGCTACAGGCATCGTGGTGTCACGCTCGTCGTTTGGTATGGCTTCATTCAGCTCCGGTTC
CCAACGATCAAGGCGAGTTACATGATCCCCCATGTTGTGCAAAAAAGCGGTTAGCTCCTTCGGTCCTCCGATCGT
TGTCAGAAGTAAGTTGGCCGCAGTGTTATCACTCATGGTTATGGCAGCACTGCATAATTCTCTTACTGTCATGCC
ATCCGTAAGATGCTTTTCTGTGACTGGTGAGTACTCAACCAAGTCATTCTGAGAATAGTGTATGCGGCGACCGAG
TTGCTCTTGCCCGGCGTCAATACGGGATAATACCGCGCCACATAGCAGAACTTTAAAAGTGCTCATCATTGGAAA
ACGTTCTTCGGGGCGAAAACTCTCAAGGATCTTACCGCTGTTGAGATCCAGTTCGATGTAACCCACTCGTGCACC
CAACTGATCTTCAGCATCTTTTACTTTCACCAGCGTTTCTGGGTGAGCAAAAACAGGAAGGCAAAATGCCGCAAA
AAAGGGAATAAGGGCGACACGGAAATGTTGAATACTCATACTCTTCCTTTTTCAATATTATTGAAGCATTTATCA
GGGTTATTGTCTCAT
```

E. coli **murein lipoprotein promoter** is in red and **ELVd** cDNA **(**C327 to G46 of AJ536613) is in black with the repeated hammerhead ribozyme domain on yellow background. cDNA coding for the **precursor of a Cpf1-based crRNA** in blue inserted between T245 and T246 of ELVd cDNA. Ribozymes self-cleavage sites are underlined. *E. coli* ribosomal **rrnC terminator** is in fuchsia. **pUC replication origin** is in gray and **ampicillin resistance gene** (inverse orientation) on gray background (**promoter** on dark gray background).

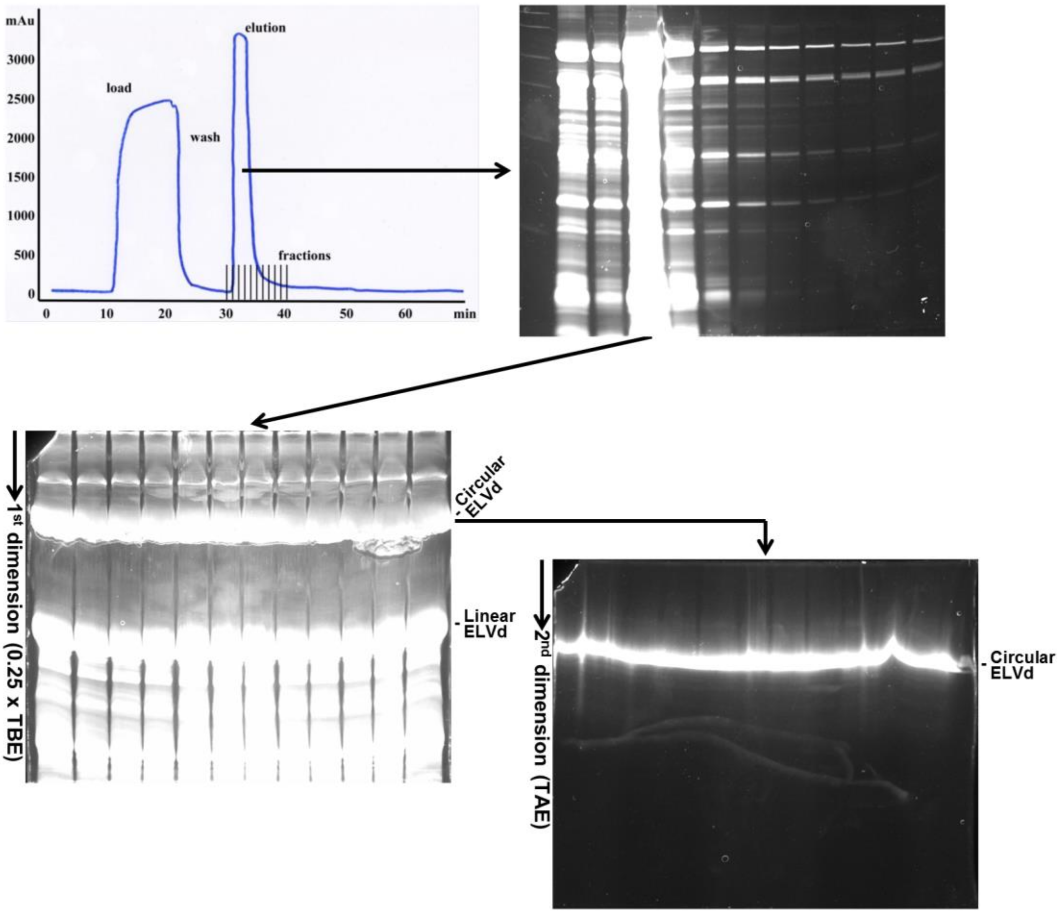

Figure S21. Workflow that illustrates the purification process of a circular RNA produced in *E. coli* using the ELVd-derived system. Total *E. coli* RNAs are first purified by anion exchange chromatography using a DEAE Sepharose column. Aliquots of the different fractions that elute in the presence of 1 M NaCl are analyzed by denaturing (8 M urea) PAGE. Selected fraction is subjected to a two-step electrophoretic separation in polyacrylamide gels. First, RNAs are separated by PAGE in 8 M urea gels in 0.25 x TBE buffer. After ethidium bromide staining of the gel, the band corresponding to the circular RNAs is excised and loaded on top of a second non-denaturing gel that is run in TAE buffer. Circular RNAs are detected in the second gel by ethidium bromide staining and eluted.
